# Using genomic relationship likelihood for parentage assignment

**DOI:** 10.1186/s12711-018-0397-7

**Published:** 2018-05-18

**Authors:** Kim E. Grashei, Jørgen Ødegård, Theo H. E. Meuwissen

**Affiliations:** 1 AquaGen AS, P.O. Box 1240, NO-7462 Trondheim, Norway; 2 Department of Animal and Aquacultural Sciences, Norwegian University of Life Sciences, P.O. Box 5003, NO-1432 Ås, Norway

## Abstract

**Background:**

Parentage assignment is usually based on a limited number of unlinked, independent genomic markers (microsatellites, low-density single nucleotide polymorphisms (SNPs), etc.). Classical methods for parentage assignment are exclusion-based (i.e. based on loci that violate Mendelian inheritance) or likelihood-based, assuming independent inheritance of loci. For true parent–offspring relations, genotyping errors cause apparent violations of Mendelian inheritance. Thus, the maximum proportion of such violations must be determined, which is complicated by variable call- and genotype error rates among loci and individuals. Recently, genotyping using high-density SNP chips has become available at lower cost and is increasingly used in genetics research and breeding programs. However, dense SNPs are not independently inherited, violating the assumptions of the likelihood-based methods. Hence, parentage assignment usually assumes a maximum proportion of exclusions, or applies likelihood-based methods on a smaller subset of independent markers. Our aim was to develop a fast and accurate trio parentage assignment method for dense SNP data without prior genotyping error- or call rate knowledge among loci and individuals. This genomic relationship likelihood (GRL) method infers parentage by using genomic relationships, which are typically used in genomic prediction models.

**Results:**

Using 50 simulated datasets with 53,427 to 55,517 SNPs, genotyping error rates of 1–3% and call rates of ~ 80 to 98%, GRL was found to be fast and highly (~ 99%) accurate for parentage assignment. An iterative approach was developed for training using the evaluation data, giving similar accuracy. For comparison, we used the Colony2 software that assigns parentage and sibship simultaneously to increase the power of the likelihood-based method and found that it has considerably lower accuracy than GRL. We also compared GRL with an exclusion-based method in which one of the parameters was estimated using GRL assignments.This method was slightly more accurate than GRL.

**Conclusions:**

We show that GRL is a fast and accurate method of parentage assignment that can use dense, non-independent SNPs, with variable call rates and unknown genotyping error rates. By offering an alternative way of assigning parents, GRL is also suitable for estimating the expected proportion of inconsistent parent–offspring genotypes for exclusion-based models.

**Electronic supplementary material:**

The online version of this article (10.1186/s12711-018-0397-7) contains supplementary material, which is available to authorized users.

## Background

In the field of animal genetics, low-density single nucleotide polymorphisms (SNPs), microsatellites, and amplified fragment length polymorphisms (AFLP) have long been the preferred types of genomic data for parentage assignment due to their low cost [1–3]. In practice, the foundation of parentage assignment rests on exclusion- and likelihood-based methods [4]. Exclusion-based methods rely on their ability to exclude false parent–offspring combinations when the offspring’s candidate parents’ genotypes violate Mendel’s laws. These methods are often used due to their ease of interpretation, but the number of expected exclusions depends on allele frequencies in the population and on genotype call rates and error rates [5]. Exclusion-based methods also require more loci than likelihood-based methods since only genotypes with Mendelian inconsistencies are used [6]. Likelihood-based methods often calculate the likelihood ratio (LR) of the genotype of the offspring, which is the probability of the offspring’s genotype given the genotypes of the candidate parents, relative to the probability of observing the genotype in the population by chance. The LR statistic effectively gives more weight to rare alleles. Different loci are typically assumed independent, such that total LR is multiplied over all loci. Likelihood-based methods have higher power than exclusion-based methods, but their interpretation is more complicated. Both likelihood- and exclusion-based models usually assume known and homogenous genotype error rates and independent loci, and do not account for variation in genotype call rates [5, 7, 8], which are all important assumptions when working with high-density SNP data. For dense SNP chip data, the assumption of independent inheritance among loci is not realistic (i.e., alleles are inherited on large DNA segments), which may lead to inflated LR values when using conventional likelihood-based methods.

Parentage can also be assigned and tested by using realized genomic relationships. The interrelationship between parents governs the expected inbreeding in offspring, as well as parent–offspring relationships. Realized genomic relationships assess the average genomic similarity across loci and do not assume independence of the loci. Increasing the number of markers in the calculations, increases the precision of the genomic relationships. Our aim was to study whether genomic relationships can be used to perform computationally fast and accurate parentage testing with high-density SNP data.

## Methods

### Residual genomic relationships

**Fig. 1:**
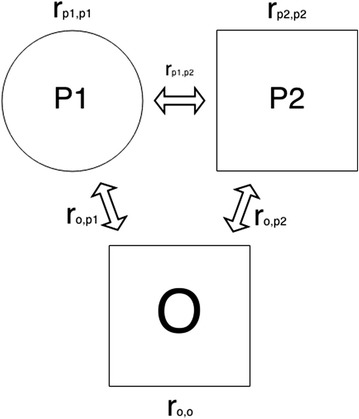
A trio of offspring (O), first parent (P1) and second parent (P2). The variables near the arrows indicate genetic relationships between individuals, while the variables over P1 and P2, and below O, are the individuals’ genetic relationships to themselves, respectively. Sexes are included in the figure but are not used by the GRL method

Estimates of genomic relationships require large numbers of loci [9], and their expectation is proportional to the genetic covariance between individuals. The proposed method for parentage testing is developed for trio parentage testing, i.e. using a single offspring and two parental candidates. The method uses genomic relationships estimated by VanRaden’s first method [10], in which the genomic relationship between two individuals is calculated as follows:

1 $$r_{ij} = \frac{{\mathop \sum \nolimits_{t = 1}^{c} \left( {m_{it} - 2p_{t} } \right)\left( {m_{jt} - 2p_{t} } \right)}}{{2\mathop \sum \nolimits_{t = 1}^{c} p_{t} \left( {1 - p_{t} } \right)}},$$

where $r_{ij}$ is the genomic relationship between individuals $i$ and $j$, $m_{it}$ and $m_{jt}$ are the genotypes (coded 0, 1 or 2 for the alternative homozygous, the heterozygous, and the homozygous reference genotypes, respectively) for individuals $i$ and $j$ at locus $t$, $p_{t}$ is the allele frequency in the population at locus $t$, and $c$ is the number of loci (i.e. SNPs). Genomic relationships can be calculated even for extremely dense genomic data (even up to full sequence), and do not assume independence of the loci. Figure 1 shows the relationships in a trio consisting of an offspring and two (candidate) parents.

We used Eq. (1) to estimate the genomic interrelationships between parents and offspring, i.e., the relationship of the offspring with itself ($r_{O,O}$), relationships of the two parent candidates with themselves ($r_{{P_{1} ,P_{1} }}$ and $r_{{P_{2} ,P_{2} }}$), relationships of the offspring with both parent candidates ($r_{{O,P_{1} }}$ and $r_{{O,P_{2} }}$), and relationships between the parent candidates ($r_{{P_{1} ,P_{2} }}$), see Fig. 1.

Expected genomic relationships of an offspring with its true parents (TP) are [11]:

$$E\left( {r_{O,P1} |TP} \right) = 0.5\left( {r_{P1,P1} + r_{P1,P2} } \right),$$

$$E\left( {r_{O,P2} |TP} \right) = 0.5\left( {r_{P2,P2} + r_{P1,P2} } \right).$$

In other words, the relationship of an offspring with a parent is the average of the genomic relationship of the parent with itself and the relationship between the two parents. The expected relationship of the offspring with itself is [12]:

$$E\left( {r_{O,O} |TP} \right) = 1 + 0.5r_{P1,P2} ,$$

where $0.5r_{P1,P2}$ is the expected inbreeding coefficient of the offspring. Three residual relationships are defined as differences between actual and expected genomic relationships:

$$e_{O,P1} = r_{O,P1} - E\left( {r_{O,P1} |TP} \right),$$

$$e_{O,P2} = r_{O,P2} - E\left( {r_{O,P2} |TP} \right),$$

$$e_{O,O} = r_{O,O} - E\left( {r_{O,O} |TP} \right).$$

Inbreeding is accounted for when using the above residuals, as well as the direction of the relationships. For example, using the offspring as a candidate parent, and/or using a true parent as the offspring, will result in large residuals, i.e., realized relationships that deviate substantially from the expectations of a true parent–offspring trio.

### Genomic relationship likelihood (GRL)

The above residual relationships are used to calculate a genomic relationship log-likelihood using a multivariate normal density function, assuming:

$${\mathbf{e}}\sim N\left( {{\varvec{\upmu}},{\varvec{\Sigma}}} \right),$$

where $${\mathbf{e}} = \left[ {\begin{array}{*{20}c} {e_{O,P1} } \\ {e_{O,P2} } \\ {e_{O,O} } \\ \end{array} } \right]$$ and $${\varvec{\upmu}} = \left[ {\begin{array}{*{20}c} {\mu_{1} } \\ {\mu_{2} } \\ {\mu_{3} } \\ \end{array} } \right]$$ is a vector of the overall means for the residuals for true parent–offspring trios. In the absence of genotyping errors, the residuals are expected to be approximately normally distributed around zero (${\mathbf{e}}\sim N\left( {0,{\varvec{\Sigma}}} \right)$, see [Additional file 1: Figure S1]. The central limit theorem states that the sum of many independently and identically distributed variates will be approximately normally distributed. The variates in Eq. (1) may be considered as originating from a common (albeit unknown) distribution, but not all are independent (i.e., the effective number of loci is lower than the actual number of loci). Still, given a substantial number of loci distributed over the entire genome (i.e., most of the loci are indeed independent), genomic relationships (summed over all variates) are still likely to approach a normal distribution (see [13], Theorem 27.4). Plotting the residual relationships for true parent–offspring trios revealed that they were approximately normally distributed [see Additional file 1: Figures S1, S2 and S3].

Since genotyping errors can occur in real data (and the expected residual relationship may thus deviate from 0), parameters of the distribution of residual relationships were estimated using an iterative method (see Section “Estimation of model parameters” below). Matrix ${\varvec{\Sigma}}$ is the 3 × 3 (co)variance matrix of the three residual variates in true parent–offspring trios and was also estimated using the iterative method. The genomic relationship likelihood (GRL) was defined as:

$${\text{GRL}} = - \frac{1}{2}\left( {{\mathbf{e}} - {\boldsymbol{\upmu}}} \right)'{\varvec{\Sigma}}^{ - 1} \left( {{\mathbf{e}} - {\boldsymbol{\upmu}}}\right),$$

which is proportional to the natural logarithm of a multivariate normal density function. Based on (iteratively assigned) parent–offspring trios, a threshold for acceptable GRL values can be defined. In this study, we assumed that a parent–offspring trio had to have a GRL value that was within the highest 99% of the known parent–offspring GRL values, thus accepting a false negative rate of 1%.

### Difference between the top two trios (${\mathbf{\Delta GRL}}$)

To reduce the false positive rate and increase the true negative rate, the value of $\Delta {\text{GRL}}$ was also assessed based on:

$$\Delta {\text{GRL}} = {\text{GRL}}_{1} - {\text{GRL}}_{2} ,$$

where ${\text{GRL}}_{1}$ $\left( {{\text{GRL}}_{2} } \right)$ is the (second) highest GRL value achieved for an offspring across all candidate parent–offspring trios. This is analogous to the Δ statistic used in Marshall et al. [7], with more details in Appendix 1.

In datasets where both parents of an offspring are present and no other relatives are available, $\Delta {\text{GRL}}$ will typically be very high, since no other realistic trio exists. When other close relatives of the offspring are included among the candidate parents, $\Delta {\text{GRL}}$ may be lower due the potential existence of multiple “likely” false parent candidates, e.g. uncles, aunts, grandparents, siblings or descendants of the offspring. High relatedness to the offspring alone is not sufficient to obtain a high value for ${\text{GRL}}_{2}$ since the method accounts for interrelationships of the whole trio. For example, if the parent candidates consist of one true parent and one full-sib of the offspring, interrelationships of the trio will typically be inconsistent because of the high relationship between the two parental candidates, although the relationships of the offspring with itself and with the parent candidates may be “normal” (these should be elevated if the relationship among the two parent candidates is high). In cases where a parent is missing but many other close relatives of the offspring are present, ${\text{GRL}}_{1}$ can, in rare cases, exceed the threshold for ${\text{GRL}}_{1}$-values, but then $\Delta {\text{GRL}}$ will typically be low, since multiple highly-related candidate parents are present. Thus, thresholds for assignment must be set for both ${\text{GRL}}_{1}$ and $\Delta {\text{GRL}}$.

### Estimation of model parameters

Estimation of the GRL-parameters, i.e. ${\varvec{\upmu}}$, ${\varvec{\Sigma}}$ and the GRL threshold, is undertaken with an iterative method which is briefly described below. The $\Delta {\text{GRL}}$ threshold was set to 6.9, which implies that the best parent pair should be at least 1000 (= e^6.9^) times more likely than the second-best parent pair. See Section 2 in Additional file 2: for more details.

#### Step 1: allele dropping

Random matings between individuals from the dataset are performed in silico to produce simulated offspring. For simplicity, all loci are assumed to be inherited independently. The simulated trios are then used to obtain initial estimates of the GRL parameters. A smaller subset of the loci may be used in this step.

#### Step 2: assignment iteration

Trios are initially assigned using the GRL method based on the parameters estimated in Step 1. The method relies on the presence of true trios (albeit unknown) in the data. Parameters ${\varvec{\upmu}}$ and ${\varvec{\Sigma}}$ are then re-estimated using the newly assigned trios from evaluation data, and then used as the basis of the next assignment iteration. Iteration stops when the number of assignments is smaller than in the previous iteration. Thus, the GRL training procedure iteratively assigns trios while (re-)estimating the GRL-parameters until no more trios can be assigned. See Section 1 in Additional file 2: for more information about the training procedure.The parameter estimates obtained in the second-to-last iteration are considered optimal. To limit the number of plausible trios to test, only individuals with a relationship larger than 0.25 with an offspring were considered as potential parents, i.e. $r_{O,P1} > 0.25$ and $r_{O,P2} > 0.25$. The GRL threshold is not re-estimated in this step.

When pre-defined parameter estimates are used, the assignment process starts without estimating parameters. This is equivalent to running only the second-to-last iteration of Step 2.

### Simulation study

A simulation study was conducted to investigate the strengths and weaknesses of the GRL method. QMSim [14] was used to produce simulated datasets. The initial size of the historical population was set to 500 and remained constant for 5000 generations to achieve mutation/drift equilibrium. In generation 5001, the population size was reduced to 300, of which 100 were males and 200 were females. Twenty chromosomes were simulated, each 1 Morgan long, and the number of SNPs was set such that approximately 54,000 SNPs (53,427 to 55,517) with a minor allele frequency higher than 0.05 existed in the population. The SNP mutation rate was set to 0.00003, assuming a recurrent mutation model (i.e. only two possible alleles exist). After the historical population, a recent population was simulated over five generations, with 1000 individuals per generation (5000 individuals in total). These were produced by random mating of 100 sires and 200 dams per generation, with one sire mated with two dams and each mating resulting in five recorded offspring. Of these, the last two generations were used in the parentage assignment tests. Fifty repetitions of the QMSim simulations were performed to produce 50 datasets. Genotype errors (1 and 3%) and call rates (80–100%) were added using a custom script written in the Python programming language, allowing both erroneous and missing genotypes among individuals, see Section 2 in Additional file 2: for more information.

The GRL method was programmed in the C++ programming language that emphasizes parallel processing. The program was run in a Linux cluster environment using multiple CPU. Tests were run using the training procedure on all (evaluation) datasets. In addition, pre-estimated parameters were obtained from some of the runs with training. The datasets were not divided into offspring and parents, and thus all true offspring and parents had the potential to be assigned parents both correctly (offspring only) and incorrectly (parents and offspring).

There are three possible outcomes of the assignment process: (1) ‘Correct’, meaning correct assignment of true parents to the unknown offspring (parents must be present), (2) ‘Incorrect’, meaning wrong candidate parents were assigned and (3) ‘No-assign’, meaning no assignment was made. These were quantified for each analysis.

### Comparison with a conventional likelihood-based method

To compare GRL with other methods, we analyzed five of the simulated datasets, arbitrarily chosen from all 50 datasets, by using the Colony2 software V2.0.6.3 [15]. Colony2 uses a likelihood-based method that jointly assigns both sibship and parentage based on a simulated annealing process [16, 17]. This increases the assignment power compared to methods that use a single unknown individual (the offspring) and one or two candidate parents. Colony2 was run using a 1% genotype error (true and assumed). In addition, the following settings were chosen: (1) do not update allele frequencies, (2) assume no inbreeding, (3) no sibship scaling, (4) no sibship prior, (5) short run length, (6) use the pairwise likelihood score (PLS) and (7) allelic dropout rate set to zero for all markers. The ‘ParentPairs’-file produced by Colony2 was used to check accuracy of assignments. Any assignments for which mother, father or both were missing, or for which the assignment probability reported by Colony2 was less than 0.5, were categorized as a “No-assign”. Suggested parent pairs with at least one incorrect parent were categorized as “Incorrect” assignments and pairs with both parent candidates correct were categorized as “Correct” assignments.

### Comparison with an exclusion-based method: the binomial exclusion method

We developed an exclusion-based method in which one of the parameters was estimated using GRL-assigned trios using custom scripts written in the R programming language. Exclusion ratios (ER) for the GRL-assigned trios were calculated ${\text{as }}$ the ratio of the number of exclusions for a trio and the number of loci for which all three individuals in the trio had called genotypes. We used a binomial distribution as a basis for the new assignments, i.e. $E\sim Bin\left( {n, p} \right)$, where $E$ is the number of trio exclusions, $n$ (number of trials) is the number of calls for the trio, and $p$ (success probability) is the median ER from the GRL assigned trios.

To limit the number of trios for binomial exclusion assignment, we used the same parent–offspring genomic relationship threshold that we used for the GRL assignments, i.e. $r_{O,P1} > 0.25$ and $r_{O,P2} > 0.25$. Assignment was done in a similar manner as with GRL, using both a confidence cutoff and a $\Delta$-score. For more information, see Section 3 in Additional file 2:. We refer to this method as the binomial exclusion method (BEM) in the text.

## Results

**Fig. 2:**
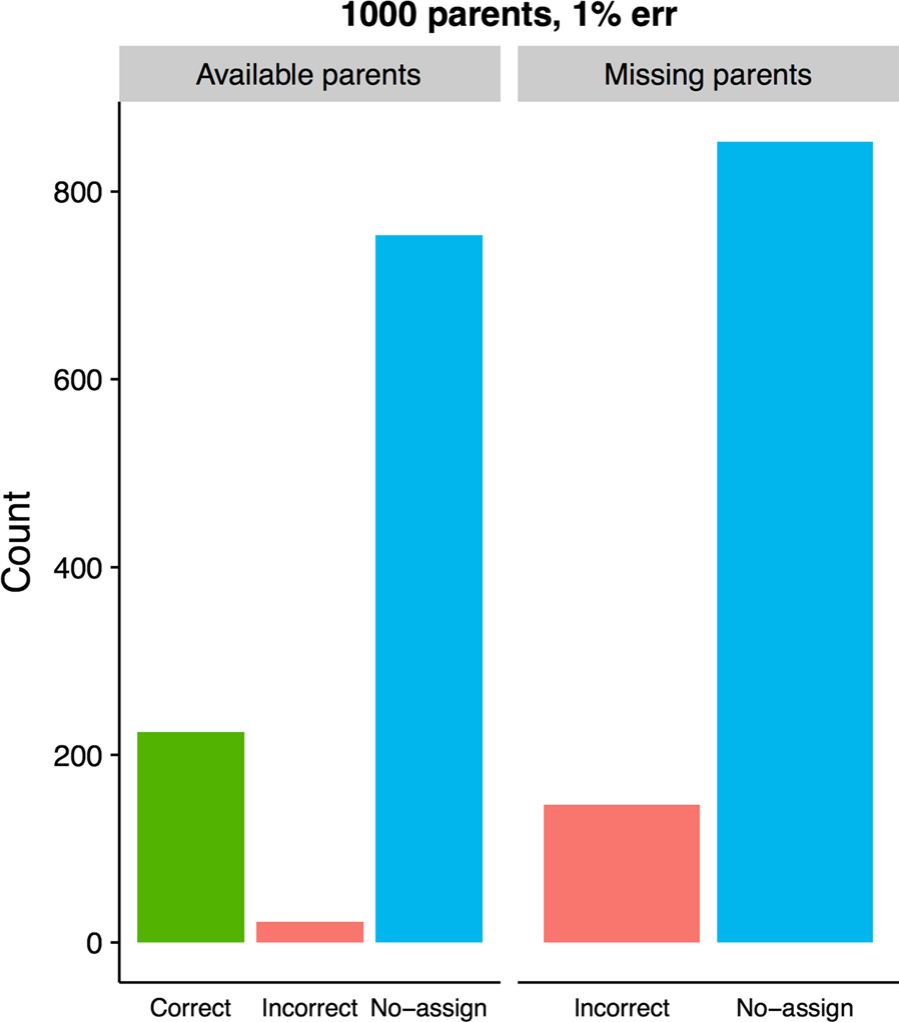
Assignment results from Colony2 for individuals with (left panel) and without (right panel) available parents in the dataset. Results from five simulated datasets are averaged. The true and assumed genotype error rate was 1% for all datasets

**Fig. 3:**
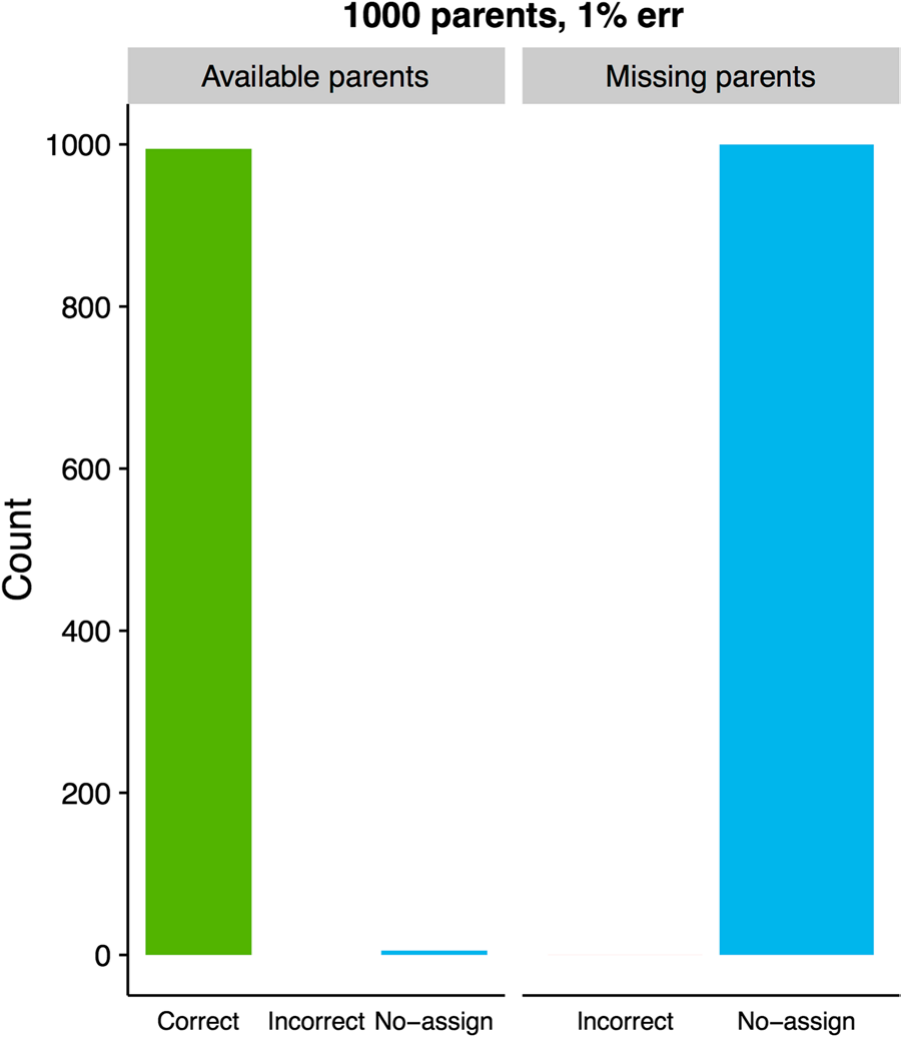
Assignment results using GRL at a 1% genotype error rate (true and assumed) for individuals with (left panel) and without (right panel) available parents in the dataset. Results from 50 simulated datasets are averaged. Parameters were pre-estimated using one arbitrarily chosen dataset with a 1% genotype error

**Fig. 4:**
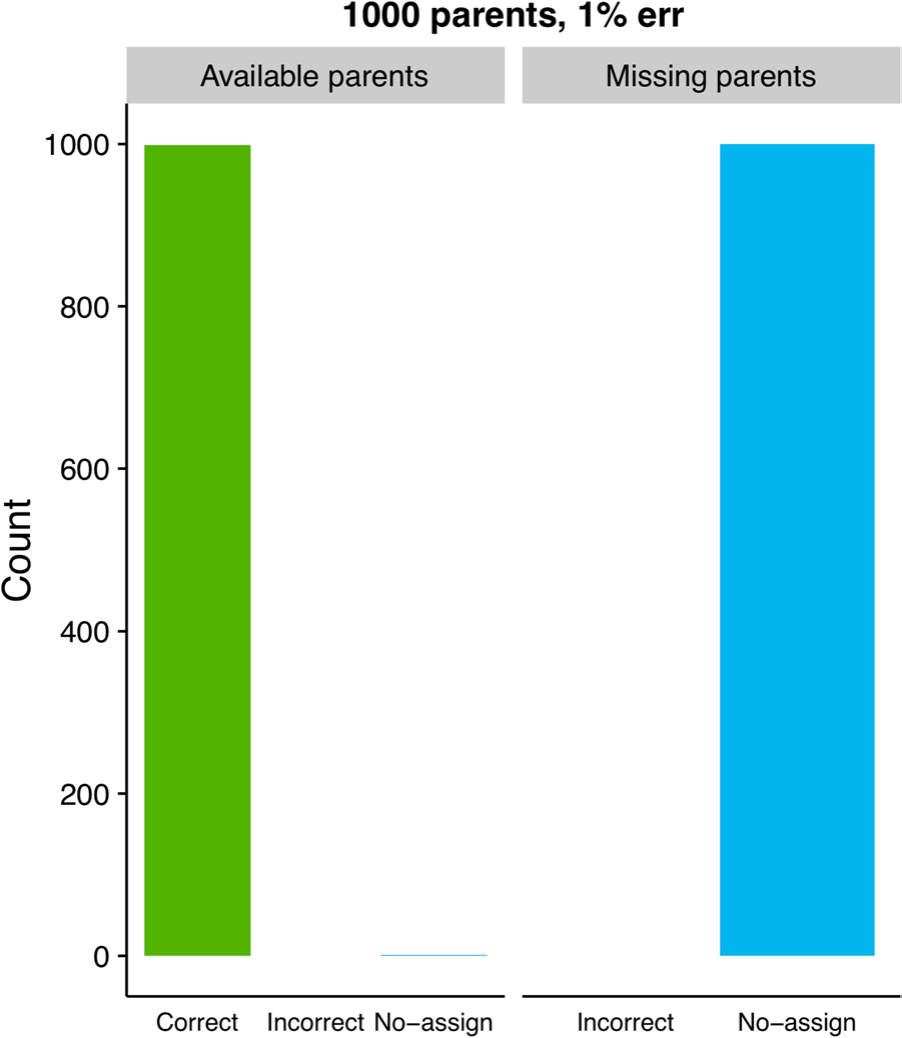
Assignment results using BEM at a 1% genotype error rate (true and assumed) for individuals with (left panel) and without (right panel) available parents in the dataset. Results from 50 simulated datasets are averaged. Parameters were pre-estimated using the GRL-assignments from one arbitrarily chosen dataset with a 1% genotype error

**Fig. 5:**
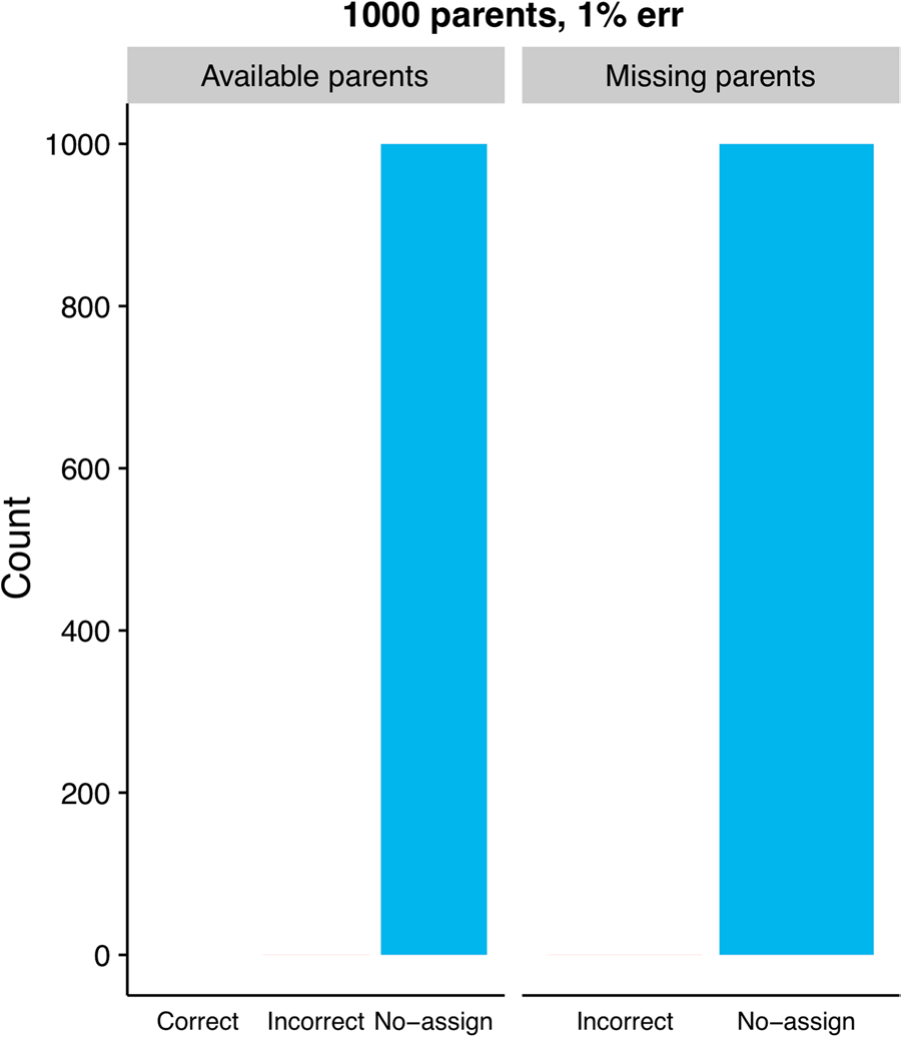
Assignment results using GRL for a 1% true genotyping error rate but using parameter estimates from a dataset with 3% genotype errors. Individuals with (left panel) and without (right panel) available parents are present in the dataset. Results from 50 simulated datasets are averaged

**Fig. 6:**
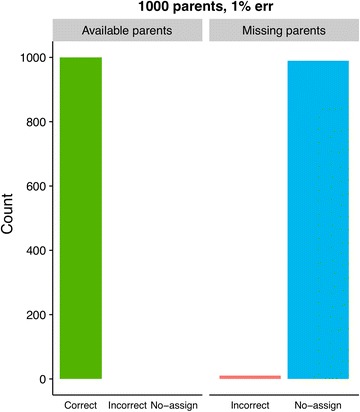
Assignment results using BEM for a 1% true genotyping error rate but using parameter estimates using GRL-assignments from a dataset with 3% genotype errors. Individuals with (left panel) and without (right panel) available parents are present in the dataset. Results from 50 simulated datasets are averaged

Assignment results using Colony2 are shown in Fig. 2, and the analogous GRL- and BEM results are shown in Figs. 3 and 4. The most noticeable differences in results between GRL- and BEM are shown in Figs. 5 and 6. Here, both methods used training estimates from a dataset with a 3% genotype error, while the true error was 1%. Results that were similar between GRL and BEM are shown in Figures S4, S5, S6, S7, S8, S9, S10 and S11 [see Additional file 3: Figures S4, S5, S6, S7, S8, S9, S10 and S11]. In Figures S4 (GRL) and S5 (BEM), parameters were pre-estimated at a 3% genotype error (true and assumed). Figures S6 (GRL) and S7 (BEM) show the results for a true error of 3% and an assumed error of 1%. Figures S8 (GRL) and S9 (BEM) show the results for training with a 1% error rate, and Figures S10 (GRL) and S11 (BEM) for training with a 3% error rate. Total results over all datasets are shown in Table S1 [see Additional file 4: Table S1].

The Colony2 software was tested using a 1% true genotype error rate (assumed and true). When parents are available, Colony2 had a correct assignment rate of 22.4%, a no-assign rate of 75.4% and an incorrect assignment rate of 2.2%. For individuals without parents, the incorrect assignment rate climbed to 14.7% and the (correct) no-assign rate climbs to 85.3% (see Fig. 2).

Figures 3 and 4 show the comparison between GRL and BEM when parameter estimates from an arbitrarily chosen dataset were used. When parents were available in the dataset and the genotype error rate (true and assumed) was 1%, using GRL resulted in 99.5% of the individuals being correctly assigned both parents (Fig. 3), while 99.9% were assigned correctly with the (GRL-trained) BEM (Fig. 4). In both cases, no individuals with parents in the dataset were assigned incorrect parent pairs. When parents were not available, the incorrect assignment rate for GRL climbed to 0.01% for both 1% and 3% genotype error rates (Fig. 3 and Additional file 3: Figure S4).

The most notable difference in results between GRL and BEM was found for a true genotype error rate of 1% when parameter estimates were from a dataset with a 3% error rate (Figs. 5 and 6). Here, GRL did not assign any trios. However, BEM assigned all trios correctly when parents were available, but incorrectly assigned 1.0% of the trios when parents were not available. When the true and assumed genotype error rates were reversed (i.e. a true error rate of 3% and an incorrectly assumed error rate of 1%), neither method assigned any trios, while the GRL method incorrectly assigned 0.02% trios, both when parents were available and when they were missing [see Additional file 3: Figures S6 and S7] and [see Additional file 4: Table S1].

An alternative to assuming a set of predefined parameters is to estimate these by using the evaluation data directly. Averaged results for each dataset are shown in Figures S8 and S9 [see Additional file 3: Figures S8 and S9] (1% genotype error) and in Figures S10 and S11 [see Additional file 3: Figures S10 and S11] (3% genotype error). These results are very similar to the results shown in Figs. 3 and 4 (1% true and assumed error rates), and Figures S4 and S5 [see Additional file 3: Figures S4 and S5] (3% true and assumed error rates).

## Discussion

Parentage assignment is mostly performed using likelihood-based models with microsatellites [2, 7], low-density SNPs [1] or exclusion-based models [18]. However, assignments methods often impose idealized assumptions, such as known age, generation and gender of all individuals, a limited number of known parental candidates, independent markers, little or no inbreeding, no stratification of the population or sample, no biased sampling of individuals, Hardy–Weinberg equilibrium (HWE) and little or no variation in genotype error or call rates within and between samples. For GRL and BEM, we perfomed assignments with unknown age, generation and gender, with no assumption as to independence of markers, HWE, inbreeding, family size or family composition, and with dense (SNP) markers, closely related individuals and varying genotype error and call rate. Colony2 assumes HWE, independent markers and no inbreeding.

### GRL

Residual relationships were approximately normally distributed even when genotype errors were present [see Additional file 1: Figures S2 and S3], but with different expectations compared to genomic data without genotype errors [see Additional file 1: Figure S1].

It did not appear to be a problem that the parent and offspring generations were unknown when using GRL and BEM. High accuracies were achieved, although individuals had numerous close relatives that were eligible as parent candidates, such as the true parents, full- and half-sibs, own offspring, uncles/aunts and nieces/nephews. Similar results were obtained when the genotype error was increased to 3%, which was used to show that the GRL and BEM work even when the genotype error rate has quite extreme values. These properties may be useful for populations with large sibling groups, such as in fish, poultry or pigs, when generations cannot be clearly differentiated, or when the genotype error or call rates vary a lot.

A strength of the GRL training procedure is that no reference dataset with known pedigree is required for training and that the training is only partly done by simulation (allele-dropping). As long as there is a sufficient number of true (but unknown) trios present for assignment, the training can proceed. The method requires a pre-defined $\Delta {\text{GRL}}$ threshold (i.e. the minimum acceptable value). The $\Delta {\text{GRL}}$ is (the log of) the odds for correct assignment, given that the correct trio is among the two best trios (this is nearly always the case if true parents are present). In this study, the threshold was set to 6.9, i.e., the best trio should be at least e^6.9^ = 1000 times more likely than the second-best trio. Relaxing this assumption will increase both the true and false positive assignment rates of the model, while setting a stricter threshold will have the opposite effect.

In some cases, the iterative training method may fail because the initial iteration results in no assignments. This may be caused by two factors: (1) the number of loci used in the allele-dropping simulation step may be set too high (giving too idealized parent–offspring relationships compared with evaluation data), or (2) there are no true trios present in the evaluation dataset. If reducing the number of SNPs used in the allele-dropping step does not start the iteration process, the latter may be the case. During training, there is no need to estimate or assume a genotype error rate with the GRL method, as long as the training procedure is done using the evaluation dataset.

Exclusion using parent–offspring duos (i.e. offspring and a single candidate parent) or trios is a relatively simple method for parentage assignment, by identifying incorrect parents by genotypes that violate the laws of Mendelian inheritance (“exclusion genotypes”). The GRL method is a fundamentally different approach and can be used to estimate exclusion-based parameters in true parent–offspring trios (assigned by GRL). Assignment of a single parent to an offspring is also possible using a similar method as for trios, but this was not explored in this study. The training-based GRL has the advantage that it requires no prior assumption with respect to genotype error rate or expected number of exclusions.

### Binomial exclusion method

Estimation of the *p*-parameter for the BEM was done using trios that were assigned using GRL. An alternative to using GRL-assignments is using a training dataset with genotyped trios and known pedigree. Such a training dataset would need to have a similar genotype error rate as the evaluation dataset since having a discrepancy between the true and assumed genotype error rate could lead to decreased accuracy [see Additional file 4: Table S1]. Since pedigree information is not always reliable, we prefer to use GRL assignments (preferably using a relatively big dataset) for parameter estimation.

### Comparing GRL and the binomial exclusion method with Colony2

The GRL and BEM resulted in much more accurate assignments of parents than Colony2. Parameters for Colony2 were chosen to minimize running time, so assignment accuracy may be improved by adjusting the parameters, but at the expense of time and/or computing resources required to perform the analysis. Colony2 incorrectly assumes that marker loci are independently distributed, while GRL and BEM do not. This is likely the main reason for the poor results obtained with Colony2 on these relatively dense marker datasets.

### Comparing GRL with the binomial exclusion method

Using BEM resulted in a slightly higher accuracy than GRL when the genotype error assumption was correct, or when GRL-parameters were estimated using the evaluation data (Figs. 3 and 4) and [see Additional file 3: Figures S4, S5, S8 and S9]. However, when pre-estimated model parameters are used, assuming a too high genotype error rate will lead to some false assignments with BEM (Fig. 6), and assignment failure for the GRL method (Fig. 5). Thus, GRL can be used when it is crucial to minimize the false-positive rate. Assuming a too low genotype error rate resulted in both methods failing to correctly assign any trios, but GRL had a small fraction (0.016%) of false assignments while BEM did not [see Additional file 4: Table S1]. Although the success parameter (*p*, see Methods) of BEM was estimated using already GRL-assigned trios, the results indicate that the two methods are somewhat complementary and can be used together to increase overall assignment accuracy.

When the assumed genotype error rate was correct (Figs. 3 and 4) and [see Additional file 3: Figures S4 and S5] or when the evaluation dataset was used to estimate parameters [see Additional file 3: Figures S8, S9, S10 and S11], nearly all the individuals were assigned correctly and there were hardly any false assignments with either method. Thus, parameters should be estimated using the available data whenever possible, which should be the case in most situations.

### Using GRL with clones or duplicated DNA

A possible novel use for the GRL method is analysis of genomic data that contain possibly duplicated genomes (e.g., by sampling of clones in plants or monozygotic twins in animals, or by duplicated sampling of DNA from the same individual). Using traditional likelihood-based or exclusion-based methods, duplicated samples/clones should be removed prior to the analysis, as these may be assigned as their own parents. For the GRL method, duplication of offspring genotypes is not a problem since GRL looks at patterns in parent–offspring relationships rather than the likelihood of each single genotype. For example, if clones of a non-inbred offspring are inserted as one or both putative parents, the GRL method would expect the offspring to be highly inbred, which will not match the observed relationship of the offspring with itself, and thus yields a low GRL value. However, duplication of parental genotypes will inevitably lead to assignment failure, since two or more trios will appear equally likely.

## Conclusions

The GRL method is a promising trio parentage assignment method which is well suited to perform parentage assignment with high accuracy on high-density SNP datasets. GRL can be applied with success on datasets with high and/or unknown genotype error rates, highly dependent marker loci, closely-related individuals, inbreeding and in some cases clones. Estimation of the GRL parameters can be done without having a pre-existing reference dataset with known parent–offspring trio combinations. In addition, GRL can be used for training of exclusion-based methods.

### Additional files

**Additonal file 1: Figures S1, S2 and S3.** Residual relationships plotted for all true trios from the 50 datasets. This file contains three figures (Figures S1, S2 and S3). Residual densities for offspring to itself (top panel), offspring to real mother (mid panel) and offspring to real father (bottom panel) are shown as a continuous line in all Figs. 50,000 values were sampled from the normal distribution using the means and variances of the residuals as parameters, shown as a dashed line in each panel. Figure S1 shows results in which there is no genotype error or call rate variance, Figure S2 in which there is 1% genotype error and a ~ 80 to 100% call rate and Figure S3 in which there is a 3% genotype error and a ~ 80 to 100% call rate.

**Additonal file 2.** Supplementary material. This file contains three sections with extended information about the GRL training procedure, call rate and genotype error simulation, and the binomial exclusion method (BEM), respectively.

**Additonal file 3: Figures S4, S5, S6, S7, S8, S9, S10 and S11.** Assignment results using GRL or BEM for individuals with (left panel) and without (right panel) available parents in the dataset. This file contains eight figures in which assignment results from 50 simulated datasets are averaged. Parameters were pre-estimated using one arbitrarily chosen dataset in Figures S4, S5, S6 and S7, while training was performed on each evaluation dataset in Figures S8, S9, S10 and S11. Figures S4, S6, S8 and S10 show results using GRL, while Figures S5, S7, S9 and S11 show results using BEM. Figures S4 and S5 show results when there is a 3% genotype error (true and assumed), Figures S6 and S7 have pre-esimated parameters from a dataset with a 1% genotype error, while the (true) evaluation genotype error is 3%. Figures S8, S9, S10 and S11 use training on each evaluation dataset, both at 1% (Figures S8 and S9) and 3% (Figures S10 and S11) genotype errors. In all figures, the call rates are ~ 80 to 100%.

**Additonal file 4: Table S1.** Summary table of total number of correct, incorrect and non-assigned trios with or without parents and genotype errors for all 50 datasets. Genotype error: either 1% or 3%, and with assumption of genotype error in parenthesis (only applicable for models that are pre-trained). Available parents: all individuals with parents available for assignment in the dataset (Yes) or where all parents are missing (No). Correct: Number of correctly assigned individuals over all 50 datasets (only applicable when parents are available). Incorrect: Number of incorrectly assigned individuals over all 50 datasets. No-assign: Number of individuals that could not be assigned parents over all 50 datasets.

## Appendix

### Mathematical foundation for the GRL method

In this article, only the hypothesis of true parents is used for the GRL method:

$$H_{1}:Both\, candidate\, parents\, are \,the \,true \,parents\, of\, the \,child.$$

We assume ${\mathbf{x}}\varvec{ }\sim N\left( {{\varvec{\upmu}}, {\varvec{\Sigma}}} \right)$ where ${\mathbf{x}}$ is the vector of residual genomic relationships, i.e. it holds the residual values for trio assignments. We define $x_{1}$ as being the most probable trio, while $x_{2}$ is the second most probable trio, that is $P\left( {x_{1} |H_{1} } \right) \ge P(x_{2} |H_{1} )$.

The difference $\Delta_{\text{GRL}} = {\text{GRL}}_{1} - {\text{GRL}}_{2}$, where ${\text{GRL}}_{1}$ and ${\text{GRL}}_{2}$ refer to the best and the second best trio candidates, respectively, can be shown to be identical to the natural logarithm of the probability of observing $x_{1}$ given $H_{1}$ divided by the probability of observing $x_{2}$ given $H_{1}$. Since ${\mathbf{x}}$ is assumed to be normally distributed, the multivariate normal probability density function used is:

$$f\left( {{\mathbf{x}} |H_{1} } \right) = \frac{1}{{\sqrt {\left( {2\pi } \right)^{3} \left| \varSigma \right|} }}e^{{ - \frac{1}{2}\left( {{\mathbf{x}} - {\boldsymbol{\upmu}}}\right)'\varSigma^{ - 1} \left( {{\mathbf{x}} - {\boldsymbol{\upmu}}}\right)}} ,$$

where ${\mathbf{x}}$ is the 3 × 1 vector of genomic residuals, ${\varvec{\upmu}}$ is 3x1 vector of expected residuals and ${\varvec{\Sigma}}$ is the 3x3 covariance matrix. If we define $\frac{{L_{1} }}{{L_{2} }} = \frac{{f(x_{1} |H_{1} )}}{{f(x_{2} |H_{1} )}}$ (i.e. how many times more likely is $x_{1}$ given $H_{1}$ compared to $x_{2}$ given $H_{1}$), we find that:

$$\frac{{f(\varvec{x}_{1} |H_{1} )}}{{f(\varvec{x}_{2} |H_{1} )}} = \frac{{\frac{1}{{\sqrt {\left( {2\pi } \right)^{3} \left| {\Sigma } \right|} }}e^{{ - \frac{1}{2}\left( {{\mathbf{x}}_{1} - {\boldsymbol{\upmu}}}\right)'{\varvec{\Sigma}}^{ - 1} \left( {\varvec{x}_{1} -\varvec{\mu}} \right)}} }}{{\frac{1}{{\sqrt {\left( {2\pi } \right)^{3} \left| {\Sigma } \right|} }}e^{{ - \frac{1}{2}\left( {{\mathbf{x}}_{2} - {\boldsymbol{\upmu}}}\right)'{\varvec{\Sigma}}^{ - 1} \left( {\varvec{x}_{2} -\varvec{\mu}} \right)}} }} = \frac{{e^{{ - \frac{1}{2}\left( {{\mathbf{x}}_{1} - {\boldsymbol{\upmu}}}\right)'{\varvec{\Sigma}}^{ - 1} \left( {{\mathbf{x}}_{1} - {\boldsymbol{\upmu}}}\right)}} }}{{e^{{ - \frac{1}{2}\left( {{\mathbf{x}}_{2} - {\boldsymbol{\upmu}}}\right)'{\varvec{\Sigma}}^{ - 1} \left( {{\mathbf{x}}_{2} - {\boldsymbol{\upmu}}}\right)}} }}.$$

If we take the natural logarithm of this ratio we get:

$$\begin{aligned} \ln \left[ {\frac{{f\left( {x_{1} |H_{1} } \right)}}{{f\left( {x_{2} |H_{1} } \right)}}} \right] & = \ln \left[ {\frac{{e^{{ - \frac{1}{2}\left( {{\mathbf{x}}_{1} - {\boldsymbol{\upmu}}}\right)'{\varvec{\Sigma}}^{ - 1} \left( {{\mathbf{x}}_{1} - {\boldsymbol{\upmu}}}\right)}} }}{{e^{{ - \frac{1}{2}\left( {{\mathbf{x}}_{2} - {\boldsymbol{\upmu}}}\right)'{\varvec{\Sigma}}^{ - 1} \left( {{\mathbf{x}}_{2} - {\boldsymbol{\upmu}}}\right)}} }}} \right] \\ & = \ln \left( {e^{{ - \frac{1}{2}\left( {{\mathbf{x}}_{1} - {\boldsymbol{\upmu}}}\right)'\varvec{\varSigma}^{ - 1} \left( {{\mathbf{x}}_{1} - {\boldsymbol{\upmu}}} \right)}} } \right) - \ln \left( {e^{{ - \frac{1}{2}\left( {{\mathbf{x}}_{2} - {\boldsymbol{\upmu}}} \right)'\varvec{\varSigma}^{ - 1} \left( {{\mathbf{x}}_{2} - {\boldsymbol{\upmu}}} \right)}} } \right) \\ & = - \frac{1}{2}\left( {{\mathbf{x}}_{1} - {\boldsymbol{\upmu}}}\right)'{\varvec{\Sigma}}^{ - 1} \left( {{\mathbf{x}}_{1} - {\boldsymbol{\upmu}}}\right){-\!\!-}\left( { - \frac{1}{2}\left( {{\mathbf{x}}_{2} - {\boldsymbol{\upmu}}}\right)'{\varvec{\Sigma}}^{ - 1} \left( {{\mathbf{x}}_{2} - {\boldsymbol{\upmu}}}\right)} \right) \\ & = {\text{GRL}}_{1} - {\text{GRL}}_{2} = \Delta_{\text{GRL}} . \\ \end{aligned}$$

The above formula shows that $\Delta_{\text{GRL}}$ has a logarithmic point probability ratio expectation. We can compare this to the $\Delta_{Marshall}$ test statistic which is defined as in [7], that is:

$${\text{LOD}} = { \ln }\left( {LR} \right) = \ln \left( {\frac{{P\left( {data |H_{1} } \right)}}{{P\left( {data |H_{2} } \right)}}} \right),$$

where $H_{2}$ is defined as:

$$H_{2}: Two \,random \,individuals\,are\,the\,true\,parents\,of\, the \,child.$$

LOD_1_ is defined to be the LOD-score of the most likely trio, while LOD_2_ is the second most likely trio. Then:

$$\Delta_{\text{Marshall}} = {\text{LOD}}_{1} - {\text{LOD}}_{2} = \ln \left( {LR_{1} } \right) - { \ln }\left( {LR_{2} } \right).$$

Both $P\left( {data |H_{1} } \right)$ and $P\left( {data |H_{2} } \right)$ can be written as follows:

$$\begin{aligned} P\left( {data |H_{1} } \right) & = \mathop \prod \limits_{t = 1}^{c} P_{t} \left( {g_{C} |g_{F} ,g_{M} ,H_{1} } \right)*P_{t} \left( {g_{F} } \right)*P_{t} \left( {g_{M} } \right), \\ P\left( {data |H_{2} } \right) & = \mathop \prod \limits_{t = 1}^{c} P_{t} \left( {g_{C} |H_{2} } \right)*P_{t} \left( {g_{F} } \right)*P_{t} \left( {g_{M} } \right), \\ \end{aligned}$$

where $P_{t} \left( {g_{C} |g_{F} ,g_{M} ,H_{1} } \right)$ is the probability of observing the offspring genotype given the father and mother genotypes under $H_{1}$ at locus *t*, $P_{t} \left( {g_{F} } \right)$ is the probability of observing the father genotype at locus $t$, $P_{t} \left( {g_{M} } \right)$ is the probability of observing the mother genotype at locus $t$, $P_{t} \left( {g_{C} |H_{2} } \right)$ is the probability of observing the offspring genotype under $H_{2}$ at locus $t$ and $c$ is the number of loci. Since $LR = \frac{{P(data|H_{1} )}}{{P(data|H_{2} )}}$, we can simplify $LR$ to be:

$$\begin{aligned} LR = \frac{{P\left( {data |H_{1} } \right)}}{{P\left( {data |H_{2} } \right)}} & = \mathop \prod \limits_{t = 1}^{c} \frac{{P_{t} \left( {g_{C} |g_{F} ,g_{M} ,H_{1} } \right)*P_{t} \left( {g_{F} } \right)*P_{t} \left( {g_{M} } \right)}}{{P_{t} \left( {g_{C} |H_{2} } \right)*P_{t} \left( {g_{F} } \right)*P_{t} \left( {g_{M} } \right)}} \\ & = \mathop \prod \limits_{t = 1}^{c} \frac{{P_{t} \left( {g_{C} |g_{F} ,g_{M} ,H_{1} } \right)}}{{P_{t} \left( {g_{C} |H_{2} } \right)}}. \\ \end{aligned}$$

Since $LR_{1}$ is the likelihood ratio of the most likely trio and $LR_{2}$ is the likelihood ratio of the second most likely trio (defined above), we can write $LR_{1}$ and $LR_{2}$ as:

$$LR_{1} = \frac{{P(data_{1} |H_{1} )}}{{P(data_{1} |H_{2} )}} = \mathop \prod \limits_{t = 1}^{c} \frac{{P_{t} \left( {g_{C} |g_{{F_{1} }} ,g_{{M_{1} }} ,H_{1} } \right)}}{{P_{t} \left( {g_{C} |H_{2} } \right)}},$$

and

$$LR_{2} = \frac{{P(data_{2} |H_{1} )}}{{P(data_{2} |H_{2} )}} = \mathop \prod \limits_{t = 1}^{c} \frac{{P_{t} \left( {g_{C} |g_{{F_{2} }} ,g_{{M_{2} }} ,H_{1} } \right)}}{{P_{t} \left( {g_{C} |H_{2} } \right)}},$$

where $g_{{F_{1} }}$ and $g_{{M_{1} }}$ are the genotypes of the father and mother in the most likely trio at locus $t$, respectively, and $g_{{F_{2} }}$ and $g_{{M_{2} }}$ are the genotypes of the father and mother at locus $t$ in the second most likely trio, respectively. Since the same offspring is used in both trios, $g_{C}$ is the same for both $LR_{1}$ and $LR_{2}$ for locus $t$.

Inserting $LR_{1}$ and $LR_{2}$ into the $\Delta_{Marshall}$-formula above we get:

$$\begin{aligned} \Delta_{Marshall} &= \ln (LR_{1} ) - \ln (LR_{2} ) = \ln \left( {\frac{{LR_{1} }}{{LR_{2} }}} \right) \\ & = \ln \left( {\frac{{\mathop \prod \nolimits_{t = 1}^{n} \frac{{P_{t} \left( {{\text{g}}_{C} | {\text{g}}_{{F_{1} }} ,{\text{g}}_{{M_{1} }} ,H_{1} } \right)}}{{P_{t} \left( {{\text{g}}_{C} |H_{2} } \right)}}}}{{\mathop \prod \nolimits_{t = 1}^{n} \frac{{P_{t} \left( {{\text{g}}_{C} | {\text{g}}_{{F_{2} }} ,{\text{g}}_{{M_{2} }} ,H_{1} } \right)}}{{P_{t} \left( {{\text{g}}_{C} |H_{2} } \right)}}}}} \right) \\ & = \ln \left( {\mathop \prod \limits_{t = 1}^{n} \frac{{P_{t} \left( {{\text{g}}_{C} | {\text{g}}_{{F_{1} }} ,{\text{g}}_{{M_{1} }} ,H_{1} } \right)}}{{P_{t} \left( {{\text{g}}_{C} | {\text{g}}_{{F_{2} }} ,{\text{g}}_{{M_{2} }} ,H_{1} } \right)}}} \right) \\ & = \ln \left( {\frac{{P\left( {{\mathbf{g}}_{C} |{\mathbf{g}}_{{F_{1} }} ,{\mathbf{g}}_{{M_{1} }} ,H_{1} } \right)}}{{P\left( {{\mathbf{g}}_{C} |{\mathbf{g}}_{{F_{2} }} ,{\mathbf{g}}_{{M_{2} }} ,H_{1} } \right)}}} \right), \\ \end{aligned}$$

where the explanation for ${\text{g}}_{C}$, ${\text{g}}_{{F_{1} }} ,{\text{g}}_{{M_{1} }}$, ${\text{g}}_{{F_{2} }} ,{\text{g}}_{{M_{2} }}$ is the same as above, while ${\mathbf{g}}_{C}$, ${\mathbf{g}}_{{F_{1} }}$, ${\mathbf{g}}_{{M_{1} }}$, ${\mathbf{g}}_{{F_{2} }}$ and ${\mathbf{g}}_{{M_{2} }}$ are the genotypes for the offspring (or child), for most probable father and mother and for the second most probable father and mother, respectively, over all loci in vector-notation. The $\Delta_{Marshall}$ method only uses the probability of observing the child genotypes given that $F_{1}$ and $M_{1}$, or $F_{2}$ and $M_{2}$ are the true parents. The fact that the information in the $H_{1}$ hypothesis is not used makes the $\Delta_{Marshall}$ method similar to $\Delta_{GRL}$, we see this when the two method definitions are compared:

$$GRL \,method:\Delta_{GRL} = \ln \left[ {\frac{{f\left( {\varvec{x}_{1} |H_{1} } \right)}}{{f\left( {\varvec{x}_{2} |H_{1} } \right)}}} \right],$$

$$Marshall\, method:\Delta_{Marshall} = \ln \left( {\frac{{P\left( {{\mathbf{g}}_{C} |{\mathbf{g}}_{{F_{1} }} ,{\mathbf{g}}_{{M_{1} }} ,H_{1} } \right)}}{{P\left( {{\mathbf{g}}_{C} |{\mathbf{g}}_{{F_{2} }} ,{\mathbf{g}}_{{M_{2} }} ,H_{1} } \right)}}} \right).$$

Both methods produce an estimated logarithmic ratio of the probability that $C$ is the child of the two most probable parent candidates versus the probability that $C$ is the child of the two second most probable parent candidates, hence the results produced by the two methods can be considered analogous.
