# Supplementary figures and images for: Using genomic relationship likelihood for parentage assignment
Source: Genet Sel Evol. 2018 May 18;50:26. doi: 10.1186/s12711-018-0397-7 (PMC5960170; doi:10.1186/s12711-018-0397-7)

Figure S1

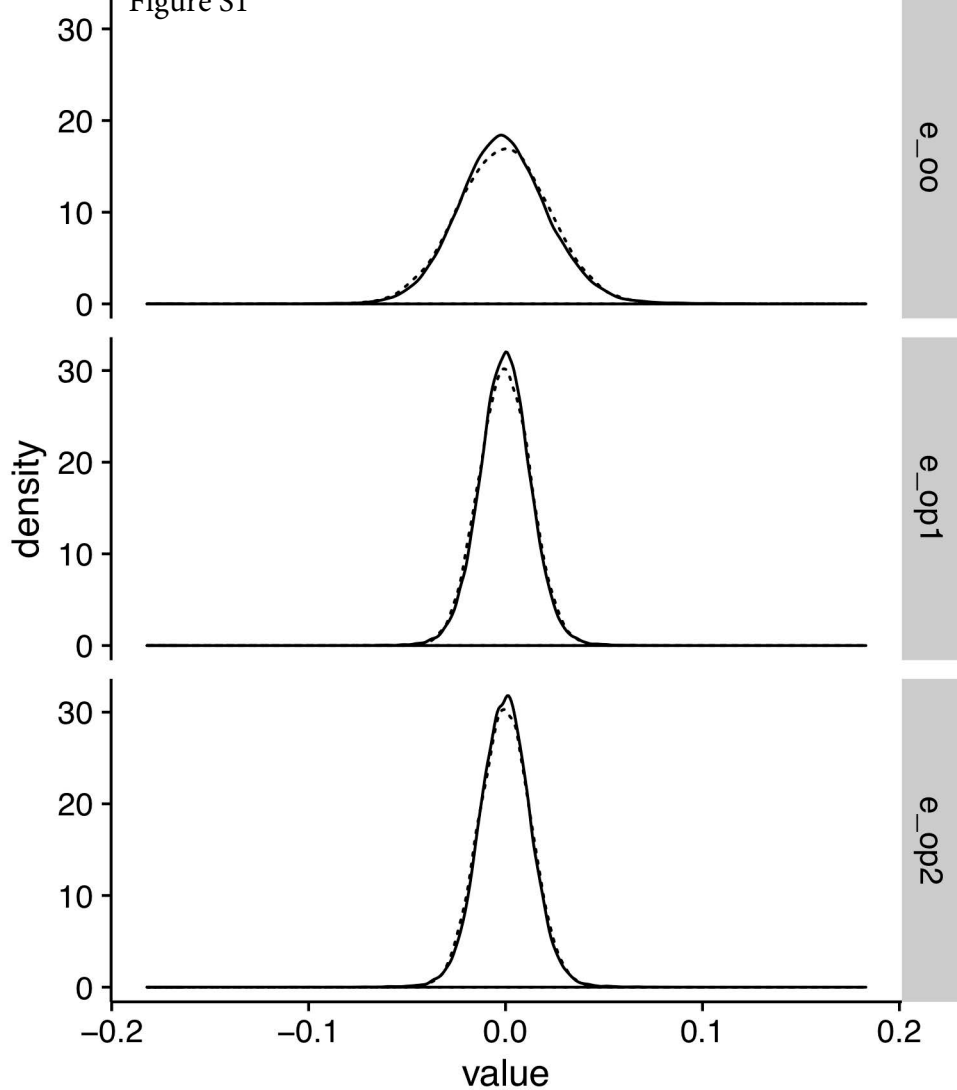

Figure S2

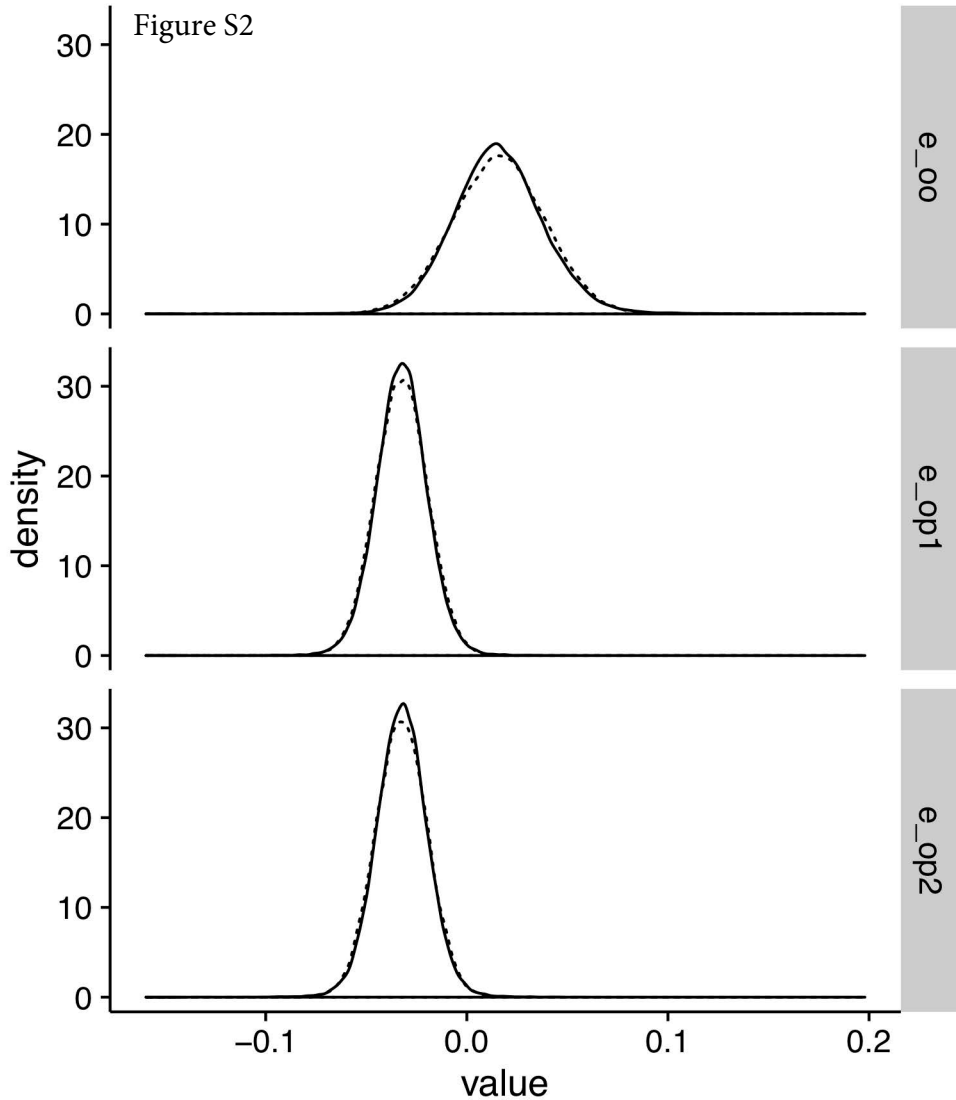

Figure S3

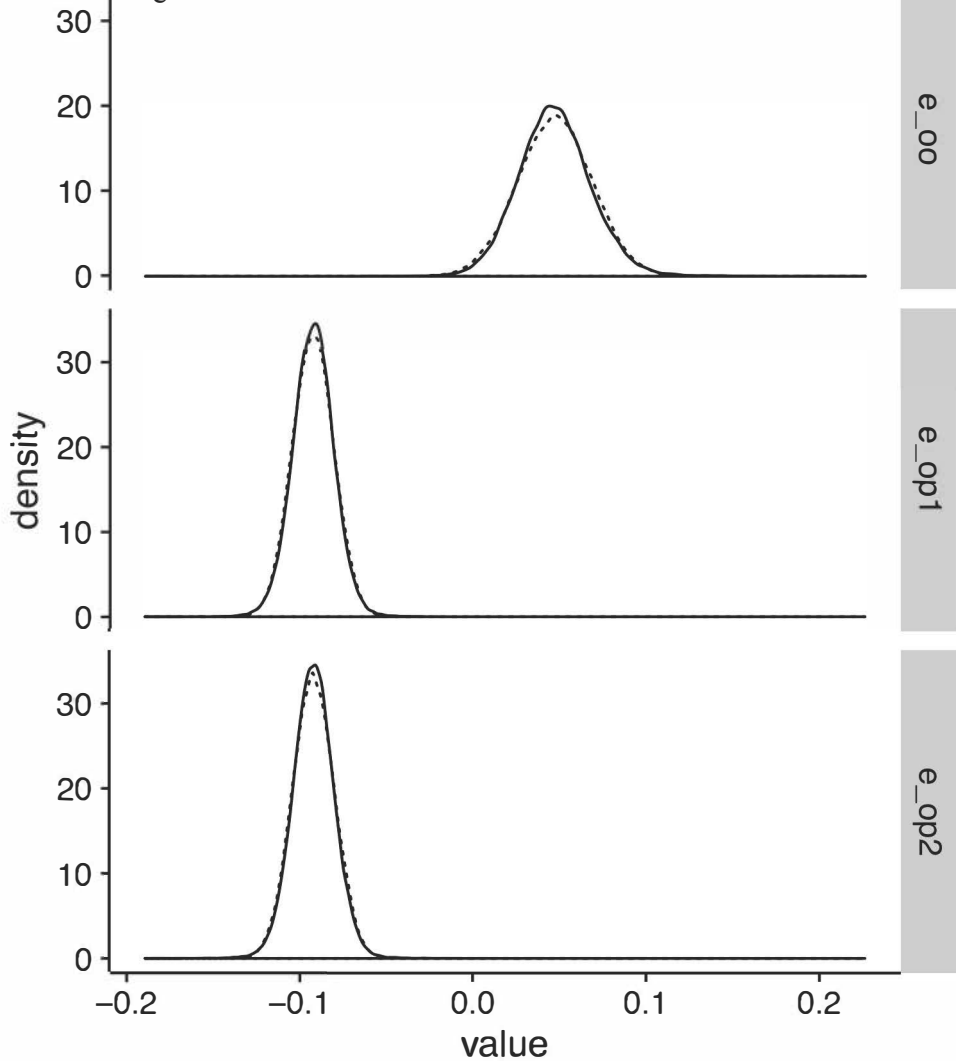

Supplement: Supplementary file 1 — Additonal file 1: Figures S1, S2 and S3. Residual relationships plotted for all true trios from the 50 datasets. This file contains three figures (Figures S1, S2 and S3). Residual densities for offspring to itself (top panel), offspring to real mother (mid panel) and offspring to real father (bottom panel) are shown as a continuous line in all Figs. 50,000 values were sampled from the normal distribution using the means and variances of the residuals as parameters, shown as a dashed line in each panel. Figure S1 shows results in which there is no genotype error or call rate variance, Figure S2 in which there is 1% genotype error and a ~ 80 to 100% call rate and Figure S3 in which there is a 3% genotype error and a ~ 80 to 100% call rate. [file 12711_2018_397_MOESM1_ESM.pdf]
