# Supplementary material for: Using genomic relationship likelihood for parentage assignment
Source: Genet Sel Evol. 2018 May 18;50:26. doi: 10.1186/s12711-018-0397-7 (PMC5960170; doi:10.1186/s12711-018-0397-7)
Supplement: Supplementary file 2 — Additonal file 2. Supplementary material. This file contains three sections with extended information about the GRL training procedure, call rate and genotype error simulation, and the binomial exclusion method (BEM), respectively. [file 12711_2018_397_MOESM2_ESM.docx]

**Additional file 2 Supplementary material**

**Section 1: Parameter estimation using allele dropping and assignment iterations**

Estimating the parameters of the GRL model, i.e. $\boldsymbol{\mu}$, $\boldsymbol{\Sigma}$ and GRL threshold, requires a dataset with a sizeable number of true trios (i.e. multiple cases of true child-parent trios). Not all children (unknown individuals) are required to have parents present in the data, as long as some true (albeit unknown) trios are present.

For a single offspring; if the real parents are present in the data, we expect the correct trio to be much more likely than all other trios. For assignment, we thus required the best trio (per offspring) to be at least 1.000 times more likely than the second-best trio, i.e., on the logarithmic scale: $\Delta GRL=\log_{e} \left( \frac{f\left( x_{1}|H_{1} \right)}{f\left( x_{2}|H_{1} \right)} \right)=\log_{e} \left( f\left( x_{1}|H_{1} \right) \right)-\log_{e} \left( f\left( x_{2}|H_{1} \right) \right)=GRL_{1}-GRL_{2}\geq\log_{e} 1000\approx6.9$, where $f(x_{i}|H_{1})$ is the normal function for residual *i* and $H_{1}$ is the hypothesis of true parents (refer to Appendix 1 for more detailed information).

Estimation of the GRL parameters involves the following steps:

*Step 1, allele dropping*: Random matings between individuals in the dataset are performed *in silico* to produce simulated offspring. For simplicity, all loci are assumed to be inherited independently. For dense marker data, this means that the *in-silico* parent-offspring relationships will have the same expectation as true parent-offspring relationships, but much smaller variance. In reality, the effective number of segments inherited from parent to offspring is rather limited (due to the limited recombination rate), and more realistic parent-offspring relationships may be achieved by using a subsample of the loci (e.g., 100 loci). The simulated trios are then used for initial estimates of the GRL parameters. Randomly mating two individuals might produce highly inbred children in some cases, an individual may even be mated to itself (i.e. selfing), but the GRL method accounts for this. The GRL assignment threshold is then chosen by ordering the in-silico generated GRL values by ascending value, and picking the value that is in the 1% location, e.g. if there are 50 000 GRL values ordered from lowest (i.e. poorest) to highest, the threshold will be the value at position 50 (50 000 * 0.01). GRL values below this threshold are considered as false trios. Initial estimates for $\boldsymbol{\mu}$ and $\boldsymbol{\Sigma}$ are obtained from the simulated parent-offspring trios. In this study, we used 50 000 in-silico matings and 100 markers for the allele-dropping step. The low number of markers used with allele-dropping is to compensate for the assumption of independence between markers.

*Step 2, assignment iteration*: Now, real data is analyzed. Unknown (but true) trios are initially assigned using the GRL method with the parameters estimated in step 1. The parameters estimated in step 1 are from idealized simulated trios, and the initial assignments will thus be highly conservative due to the low variance estimates obtained from allele dropping. Parameters $\boldsymbol{\mu}$ and $\boldsymbol{\Sigma}$ are then re-estimated using the newly assigned trios from real data, and then used as the basis of the next assignment iteration. When all (or most) true trios have been included in the iteration assignment there are two possibilities; (1) no further trios are assigned due to the difference in GRL values between the worst true trio and the best false trio and the iteration stops, or (2) false trios (i.e. trios where the parents are incorrect) are assigned and included in the parameter estimation for the next iteration, effectively increasing the residual variances drastically. Due to the increase of variance estimates in (2), false trios will start to seem plausible, and the $\Delta GRL$ values will decrease, effectively reducing the number of assignments. Because of this behavior, the iteration process ceases if the number of assignments in iteration *n* is equal or less that of iteration *n-1*. Final parameter estimates from the iteration process are always from iteration *n-1* (the iteration with most assignments).

An apparent problem may arise when there are few or no true trios to estimate the GRL parameters in the initial round of step 2. In this case, it is possible to produce GRL values using the parameter estimates from step 1, but this will result in very restrictive assignments with few or no false positives and many false negatives if many genetic markers are used when performing allele dropping. This is because the allele dropping method used in step 1 assumes independent markers, which results in very low residual variance, again resulting in restrictive assignments when used on real data where the markers are clearly not independent, underlining the necessity to run the step 2 iterations to re-estimate the parameters. A possible solution is to use fewer markers when performing allele dropping in step 1. Another solution is to use pre-defined parameter estimates from a previous training session where the genotype errors and call rates are similar to the ones where training cannot be performed.

**Section 2: Call rates and genotype errors**

The locus-specific call rates were simulated as:

$$\varepsilon_{l}=1-\frac{x_{l}}{\max\left( x \right)}$$

where $x_{l}$ is a randomly sampled value from a chi-square distribution with one degree of freedom, and $\max(x)$ is the maximum value produced from the chi-square distribution over all loci. The call rate function above was found to closely resemble the shape of actual call rate distributions in real datasets. For this function, the overall call rate over (54k) loci is ~0.94-0.95.

In real data, the call rates may vary among individuals, depending on DNA quality and other factors. In such cases, the call rate sampled above may be viewed as an overall locus-specific call rate, while individual call rates may deviate from this. To mimic such situations, individuals were randomly assigned into three groups of call rates; ‘Good’ (70% of the individuals), ‘Fair’ (20% of the individuals) and ‘Poor’ (10% of the individuals). These groups were defined to have expected call rates over all loci $\left( cr_{g} \right)$ of 98%, 90% and 80%, respectively, and the expected overall call rate is then:

$$E\left( cr_{g} \right)=0.80*0.10+0.90*0.20+0.98*0.70=0.946\approx E(\varepsilon_{l})$$

i.e., the average call rate in the population is assumed approximately as simulated above (albeit higher or lower for some groups). Accordingly, different locus-specific call rates were generated for the three groups;

$$cr_{poor, l}=0.80*cr_{adj,l}=\frac{0.80*\varepsilon_{l}}{E\left( cr_{g} \right)}$$

$$cr_{fair, l}=0.90*cr_{adj,l}=\frac{0.90*\varepsilon_{l}}{E\left( cr_{g} \right)}$$

$$cr_{good, l}=0.98*cr_{adj,l}=\frac{0.98*\varepsilon_{l}}{E\left( cr_{g} \right)}$$

where $cr_{adj,l}=\frac{\varepsilon_{l}}{E\left( cr_{g} \right)}$ (i.e. the call rate at locus *l* adjusted for the population call rate expectation). Hence, the call rates of the ‘Poor’ and ‘Fair’ groups are adjusted downwards, while the call rates of the ‘Good’ group are adjusted upwards. For some loci, this may give $cr_{good, l}>1$. In such cases $cr_{good, l}$ was set to 1, resulting in an average call rate slightly lower than 0.98 for this group. For an individual genotype at locus *l*, call/no-call was sampled using the call probability given by $cr_{group,l}$, where ‘group’ is either ‘Poor’, ‘Fair’ or ‘Good’.

Subsequently, genotype errors were randomly added (for calls) at either 1% or 3% (depending on simulation scenario). As a SNP locus genotype can take three possible values, genotyping errors were randomly sampled from the two possible erroneous genotypes, assuming equal probability for each of them. Genotype errors were randomly distributed over called loci.

**Section 3: binomial exclusions – method**

Due to unknown call rate, we opted to go for a binomial distribution of number of exclusions instead of trying to find a fixed exclusion threshold. This was done in the same manner as for GRL, i.e. for 50 datasets with genotype error rates at 1% and 3%. For each dataset, we used trios already assigned by GRL to estimate the parameters for the binomial distribution:

$$E\sim Bin(n, p)$$

where *n* (number of trials) is the number of calls for the trio that is to be assigned and *p* (success probability) is the median exclusion ratio (ER) from the GRL-assigned trios. ER is calculated as:

$$ER=\frac{\#exclusions}{\#calls}$$

where $\#exclusions$ is the number of exclusions for a GRL-assigned trio and $\#calls$ is the number of called genotypes (i.e. opposite of no-calls). Only loci where all three individuals of the trio have called genotypes are used. We use median instead of mean ER to avoid possible false positive GRL-assignments affecting the estimate of *p* too much.

For each offspring, as with the GRL, trios are formed and pitted against each other. The confidence value of a binomial trio assignment is defined as $1-cdf(E)$, where $cdf$ is the cumulative distribution function. As with GRL, the two best trios are used to assign with two requirements; (1) the top trio must have a confidence value above 0.001, and (2) the $\Delta E$, i.e. the best trio likelihood divided by second-best trio likelihood, must be above a given threshold. Here, we used a $\Delta E$ threshold of 1000, i.e. the same as the $\Delta GRL$ threshold.

For each offspring, trios were formed with candidate parents having a genomic relationship above 0.25 with the offspring. This is done primarily to reduce the numbers of unlikely trios to test. This is the same threshold used as when assigning using GRL.
