# Supplementary material for: Using genomic relationship likelihood for parentage assignment
Source: Genet Sel Evol. 2018 May 18;50:26. doi: 10.1186/s12711-018-0397-7 (PMC5960170; doi:10.1186/s12711-018-0397-7)
Supplement: Supplementary file 3 — Additonal file 3: Figures S4, S5, S6, S7, S8, S9, S10 and S11. Assignment results using GRL or BEM for individuals with (left panel) and without (right panel) available parents in the dataset. This file contains eight figures in which assignment results from 50 simulated datasets are averaged. Parameters were pre-estimated using one arbitrarily chosen dataset in Figures S4, S5, S6 and S7, while training was performed on each evaluation dataset in Figures S8, S9, S10 and S11. Figures S4, S6, S8 and S10 show results using GRL, while Figures S5, S7, S9 and S11 show results using BEM. Figures S4 and S5 show results when there is a 3% genotype error (true and assumed), Figures S6 and S7 have pre-esimated parameters from a dataset with a 1% genotype error, while the (true) evaluation genotype error is 3%. Figures S8, S9, S10 and S11 use training on each evaluation dataset, both at 1% (Figures S8 and S9) and 3% (Figures S10 and S11) genotype errors. In all figures, the call rates are ~ 80 to 100%. [file 12711_2018_397_MOESM3_ESM.pdf]

Figure S4

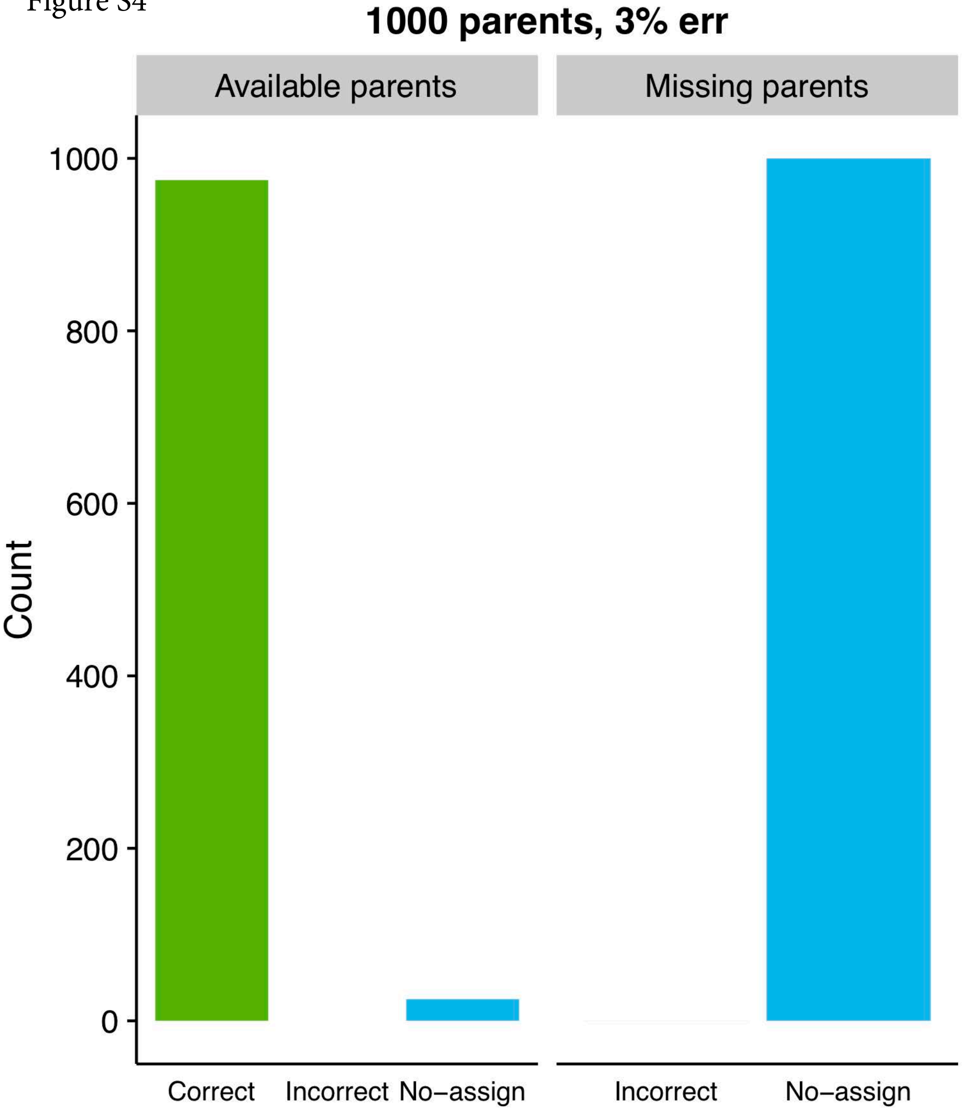
1000 parents, 3% err
Available parents
Missing parents
Count
1000
800
600
400
200
0
Correct
Incorrect
No−assign
Incorrect
No−assign

Figure S5

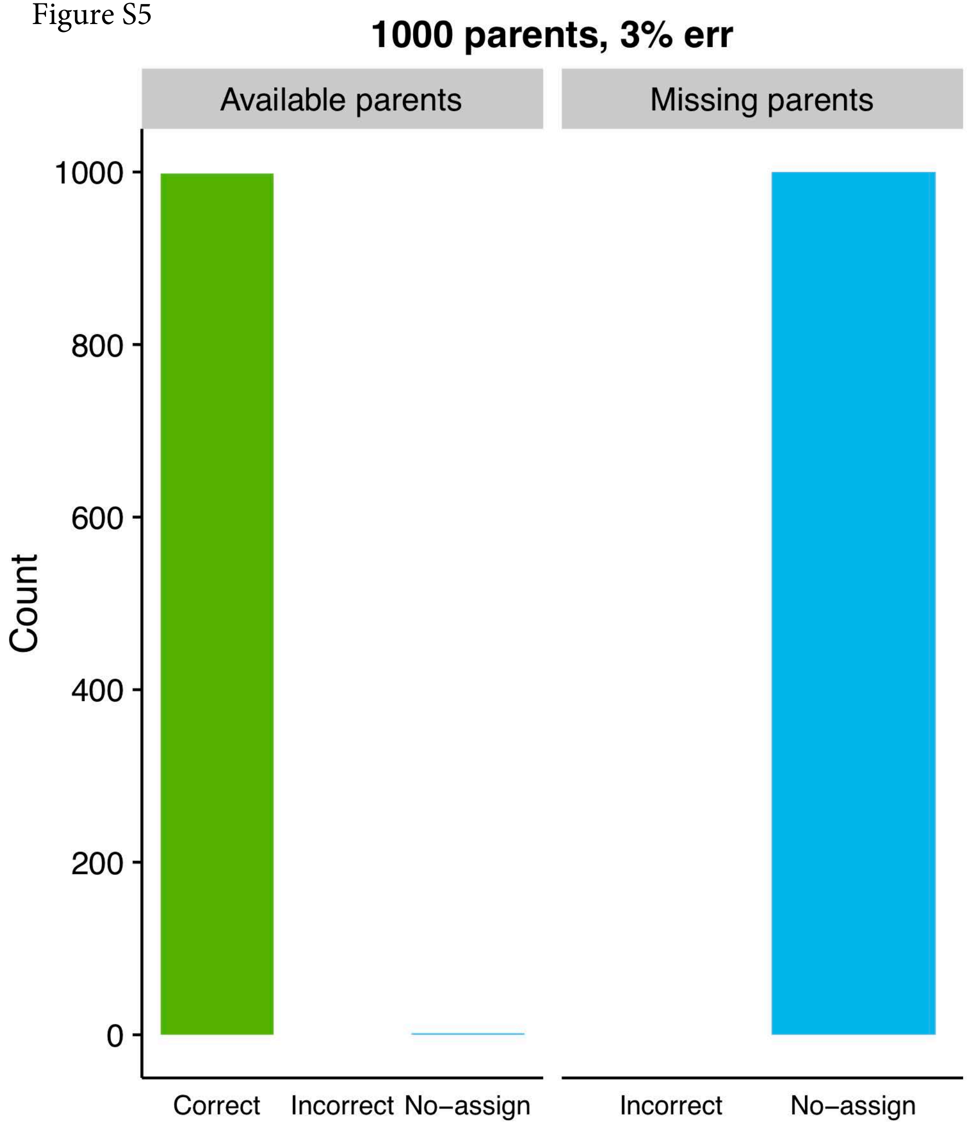
1000 parents, 3% err
Available parents
Missing parents
Count
1000
800
600
400
200
0
Correct
Incorrect
No−assign
Incorrect
No−assign

Figure S6

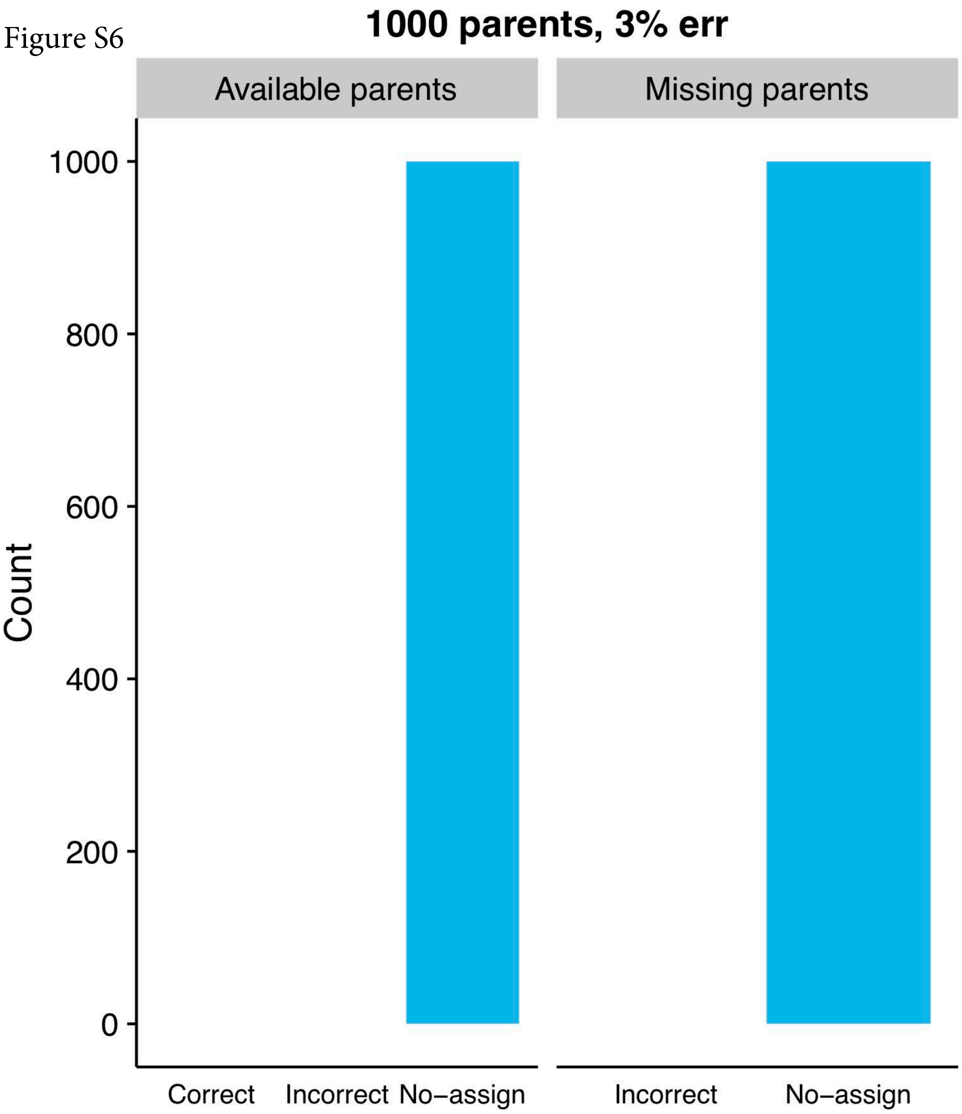
1000 parents, 3% err
Available parents
Missing parents
Count
1000
800
600
400
200
0
Correct
Incorrect
No−assign
Incorrect
No−assign

Figure S7

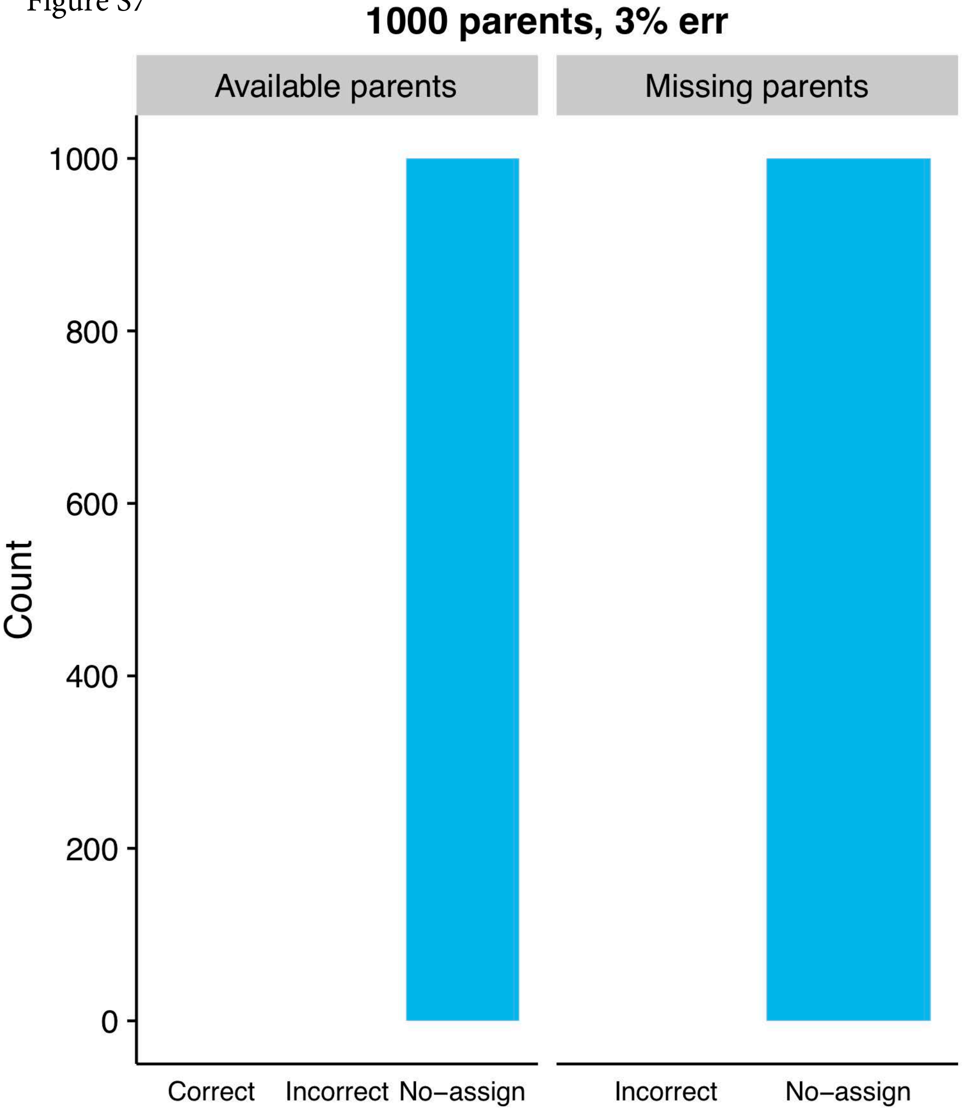
1000 parents, 3% err
Available parents
Missing parents
Count
1000
800
600
400
200
0
Correct
Incorrect
No−assign
Incorrect
No−assign

Figure S8

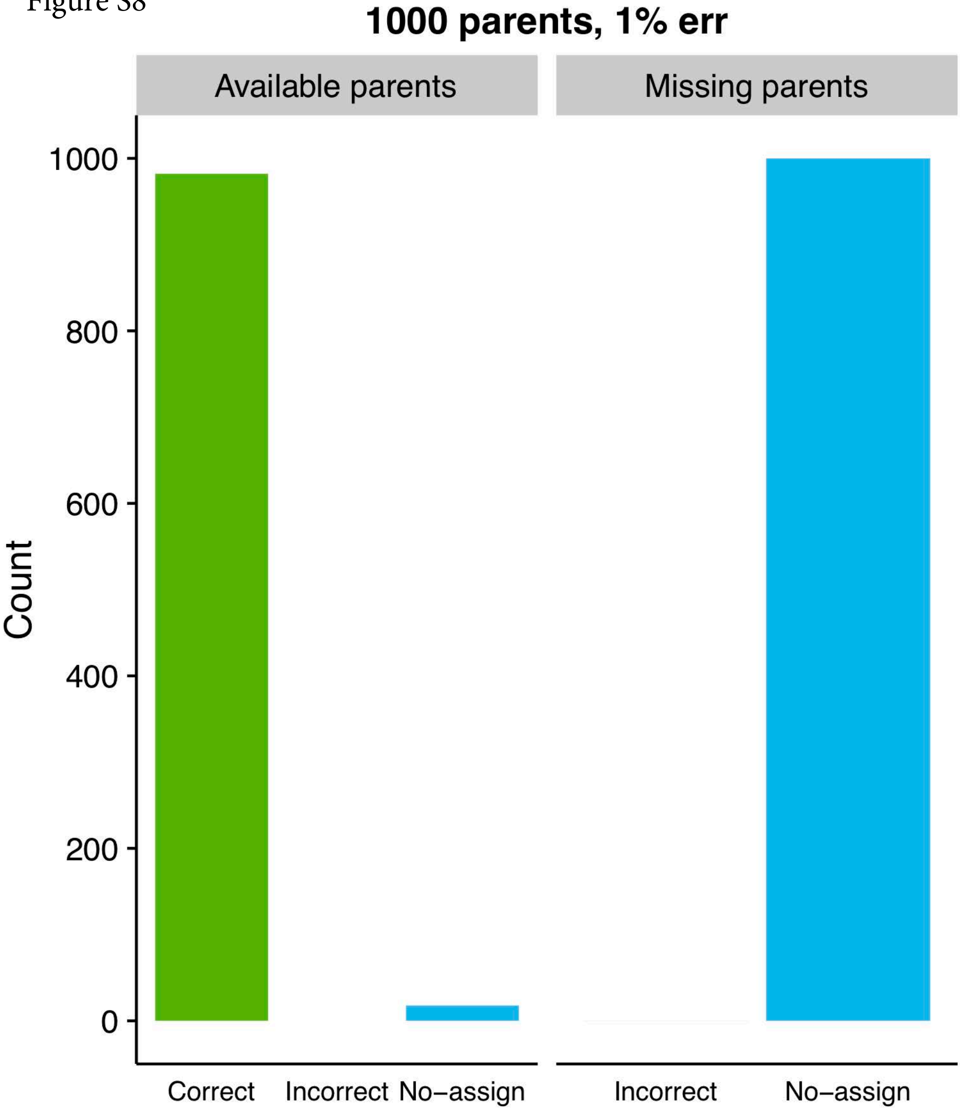
1000 parents, 1% err
Available parents
Missing parents
Count
1000
800
600
400
200
0
Correct
Incorrect
No−assign
Incorrect
No−assign

Figure S9

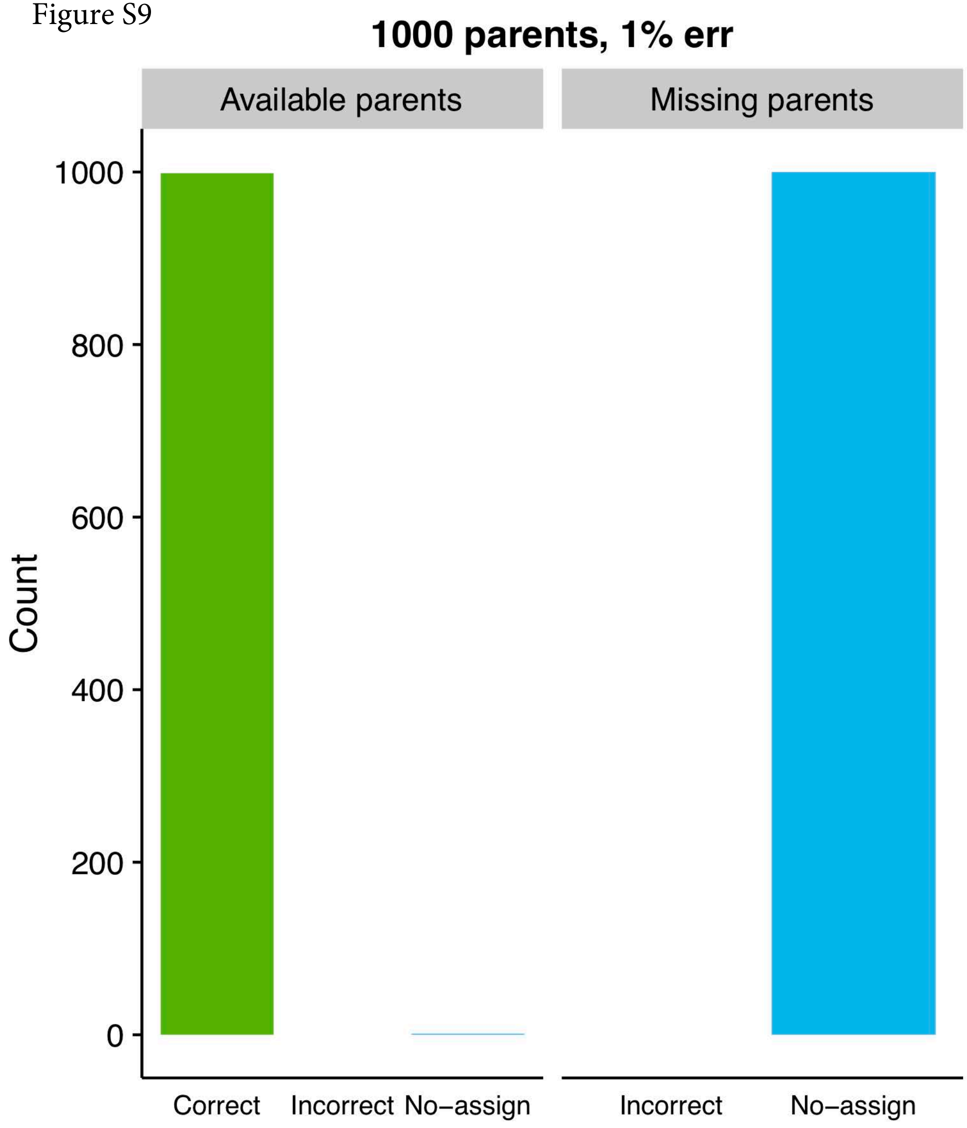
1000 parents, 1% err
Available parents
Missing parents
1000
800
600
400
200
0
Count
Correct
Incorrect
No−assign
Incorrect
No−assign

Figure S10

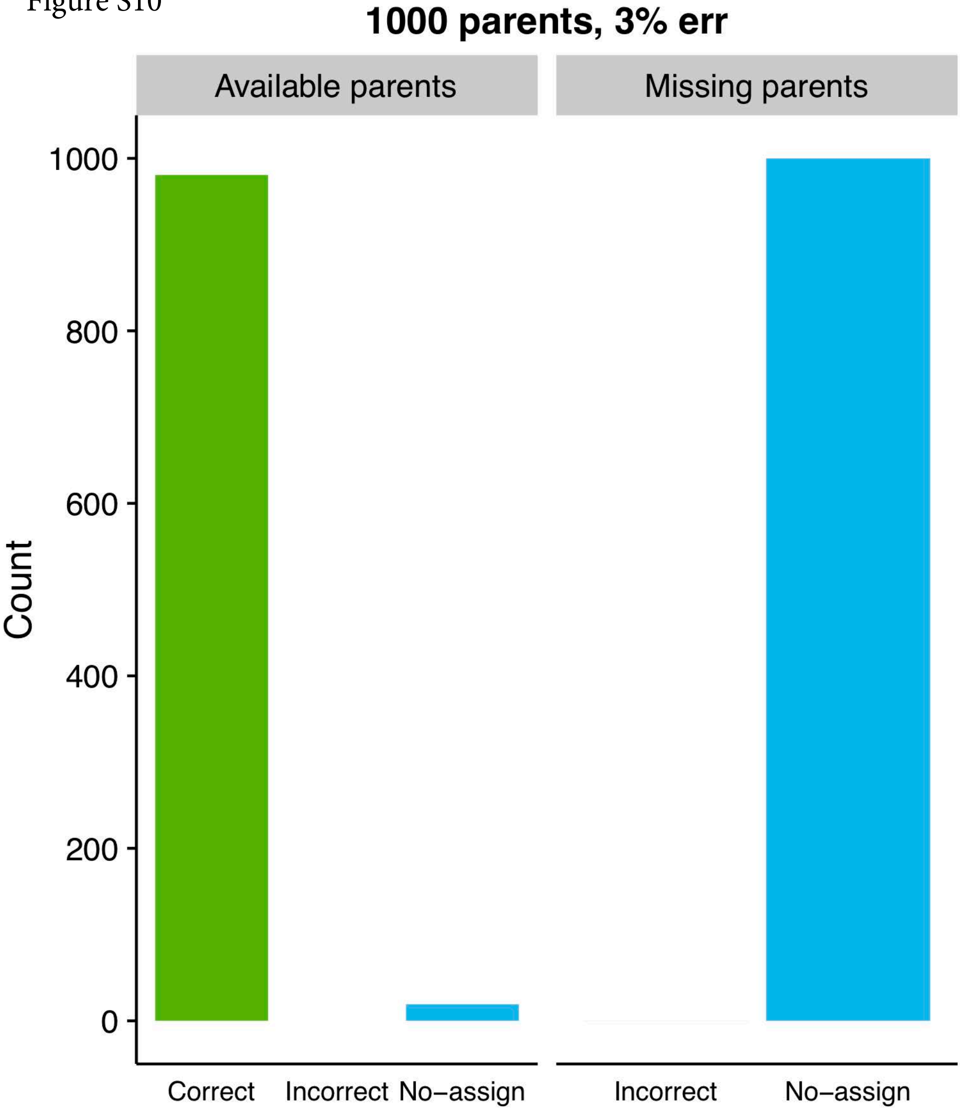
1000 parents, 3% err
Available parents
Missing parents
Count
1000
800
600
400
200
0
Correct
Incorrect
No−assign
Incorrect
No−assign

Figure S11

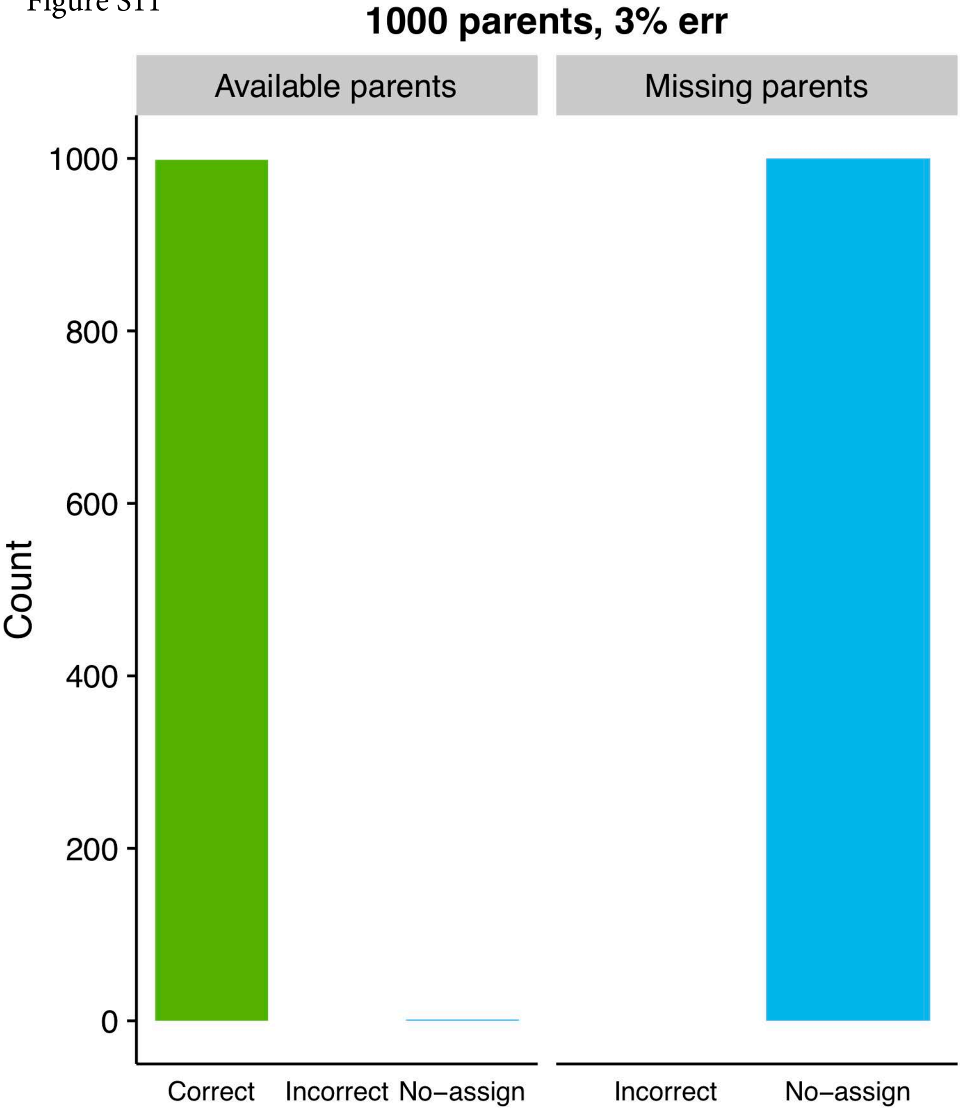
1000 parents, 3% err
Available parents
Missing parents
Count
1000
800
600
400
200
0
Correct
Incorrect
No−assign
Incorrect
No−assign
