# Supplementary material for: Using genomic relationship likelihood for parentage assignment
Source: Genet Sel Evol. 2018 May 18;50:26. doi: 10.1186/s12711-018-0397-7 (PMC5960170; doi:10.1186/s12711-018-0397-7)
Supplement: Supplementary file 4 — Additonal file 4: Table S1. Summary table of total number of correct, incorrect and non-assigned trios with or without parents and genotype errors for all 50 datasets. Genotype error: either 1% or 3%, and with assumption of genotype error in parenthesis (only applicable for models that are pre-trained). Available parents: all individuals with parents available for assignment in the dataset (Yes) or where all parents are missing (No). Correct: Number of correctly assigned individuals over all 50 datasets (only applicable when parents are available). Incorrect: Number of incorrectly assigned individuals over all 50 datasets. No-assign: Number of individuals that could not be assigned parents over all 50 datasets. [file 12711_2018_397_MOESM4_ESM.docx]

| **Method** | **Training** | **Genotype error** | **Available parents** | **Correct** | **Incorrect** | **No-assign** |
| --- | --- | --- | --- | --- | --- | --- |
| Colony2 | No | 1% (1%) | Yes | 1122 (22.44%) | 110 (2.2%) | 3768 (75.36%) |
| Colony2 | No | 1% (1%) | No | NA | 734 (14.68%) | 4,266 (85.32%) |
| GRL | No | 1% (1%) | Yes | 49,727 (99.454%) | 0 (0%) | 273 (0,546%) |
| Ex | No | 1% (1%) | Yes | 49,939 (99.88%) | 0 (0%) | 61 (0.12%) |
| GRL | No | 1% (1%) | No | NA | 5 (0.01%) | 49,995 (99,99%) |
| Ex | No | 1% (1%) | No | NA | 0 (0%) | 50,000 (100%) |
| GRL | No | 3% (3%) | Yes | 48,740 (97.48%) | 0 (0%) | 1,260 (2.52%) |
| Ex | No | 3% (3%) | Yes | 49,912 (99.82%) | 0 (0%) | 88 (0.18%) |
| GRL | No | 3% (3%) | No | NA | 5 (0.01%) | 49,995 (99.99%) |
| Ex | No | 3% (3%) | No | NA | 0 (0%) | 50,000 (100%) |
| GRL | No | 1% (3%) | Yes | 0 (0%) | 0 (0%) | 50,000 (100%) |
| Ex | No | 1% (3%) | Yes | 50,000 (100%) | 0 (0%) | 0 (0%) |
| GRL | No | 1% (3%) | No | NA | 0 (0%) | 50,000 (100%) |
| Ex | No | 1% (3%) | No | NA | 521 (1.04%) | 49,479 (98.96%) |
| GRL | No | 3% (1%) | Yes | 0 (0%) | 8 (0.016%) | 49,992 (99.984%) |
| Ex | No | 3% (1%) | Yes | 0 (0%) | 0 (0%) | 50,000 (100%) |
| GRL | No | 3% (1%) | No | NA | 8 (0.016%) | 49,992 (99.984%) |
| Ex | No | 3% (1%) | No | NA | 0 (0%) | 50,000 (100%) |
| GRL | Yes | 1% | Yes | 49,110 (98.22%) | 0 (0%) | 890 (1.78%) |
| Ex | Yes | 1% | Yes | 49,933 (99.87%) | 0 (0%) | 67 (00.13%) |
| GRL | Yes | 1% | No | NA | 4 (0.008%) | 49,996 (99.992%) |
| Ex | Yes | 1% | No | NA | 0 (0%) | 50,000 (100%) |
| GRL | Yes | 3% | Yes | 49,040 (98.08%) | 0 (0%) | 960 (1.92%) |
| Ex | Yes | 3% | Yes | 49,919 (99.84%) | 0 (0%) | 81 (0.16%) |
| GRL | Yes | 3% | No | NA | 5 (0.01%) | 49,995 (99.99%) |
| Ex | Yes | 3% | No | NA | 0 (0%) | 50,000 (100%) |

Table S1
